# The early reduction of left ventricular mass after sleeve gastrectomy depends on the fall of branched-chain amino acid circulating levels

**DOI:** 10.1016/j.ebiom.2022.103864

**Published:** 2022-02-04

**Authors:** Lidia Castagneto-Gissey, Giulia Angelini, Geltrude Mingrone, Elena Cavarretta, Leonardo Tenori, Cristina Licari, Claudio Luchinat, Anna Luise Tiepner, Nicola Basso, Stefan R. Bornstein, Deepak L. Bhatt, Giovanni Casella

**Affiliations:** a Department of Surgical Sciences, Sapienza University of Rome, Viale Regina Elena, 324, Rome, Italy; b Università Cattolica del Sacro Cuore, Rome 00161, Italy; c Fondazione Policlinico Universitario Agostino Gemelli IRCCS, Rome, Italy; d Division of Diabetes and Nutritional Sciences, Faculty of Life Sciences and Medicine, King's College London, London, United Kingdom; e Department of Medical-Surgical Sciences and Biotechnologies, Sapienza University of Rome, Latina, Italy; f Mediterranea Cardiocentro, Napoli, Italy; g Department of Chemistry, University of Florence, Italy; h Magnetic Resonances Center (CERM), University of Florence, Italy; i Department of Medicine III, Universitätsklinikum Carl Gustav Carus an der Technischen Universität Dresden, Dresden, Germany; j Brigham and Women's Hospital Heart and Vascular Center, Harvard Medical School, Boston, MA, United States

**Keywords:** Bariatric/metabolic surgery, Left ventricular mass, Epicardial fat, Metabolomics, Gene expression

## Abstract

**Introduction:**

Body-mass index is a major determinant of left-ventricular-mass (LVM). Bariatric-metabolic surgery (BMS) reduces cardiovascular mortality. Its mechanism of action, however, often encompasses a weight-dependent effect. In this translational study, we aimed at investigating the mechanisms by which BMS leads to LVM reduction and functional improvement.

**Methods:**

Twenty patients (45.2 ± 8.5years) were studied with echocardiography at baseline and at 1,6,12 and 48 months after sleeve-gastrectomy (SG). Ten Wistar rats aged 10-weeks received high-fat diet ad libitum for 10 weeks before and 4 weeks after SG or sham-operation. An oral-glucose-tolerance-test was performed to measure whole-body insulin-sensitivity. Plasma metabolomics was analysed in both human and rodent samples. RNA quantitative Real-Time PCR and western blots were performed in rodent heart biopsies. The best-fitted partial-least-square discriminant-analysis model was used to explore the variable importance in the projection score of all metabolites.

**Findings:**

Echocardiographic LVM (-12%,-23%,-28% and -43% at 1,6,12 and 48 months, respectively) and epicardial fat decreased overtime after SG in humans while insulin-sensitivity improved. In rats, SG significantly reduced LVM and epicardial fat, enhanced ejection-fraction and improved insulin-sensitivity compared to sham-operation. Metabolomics showed a progressive decline of plasma branched-chain amino-acids (BCAA), alanine, lactate, 3-OH-butyrate, acetoacetate, creatine and creatinine levels in both humans and rodents.

Hearts of SG rats had a more efficient BCAA, glucose and fatty-acid metabolism and insulin signaling than sham-operation. BCAAs in cardiomyocyte culture-medium stimulated lipogenic gene transcription and reduced mRNA levels of key mitochondrial β-oxidation enzymes promoting lipid droplet accumulation and glycolysis.

**Interpretation:**

After SG a prompt and sustained decrease of the LVM, epicardial fat and insulin resistance was found. Animal and *in vitro* studies showed that SG improves cardiac BCAA metabolism with consequent amelioration of fat oxidation and insulin signaling translating into decreased intra-myocytic fat accumulation and reduced lipotoxicity.

**Research in context**

**Evidence before this study**

Several studies have shown the beneficial effects of weight loss on cardiac function and remodeling as assessed by echocardiography, although there is a paucity of literature data regarding prospectively evaluated long-term results. Bariatric/metabolic surgery currently represents the sole modality treatment deemed to be effective in generating a substantial and durable weight loss. Moreover, bariatric/metabolic surgery shows early metabolic effects before a meaningful weight loss in type 2 diabetes although no data on cardiac function and remodeling have been investigated early after surgery.

**Added value of this study**

In this translational study, we demonstrated a net improvement of cardiac function starting just 1 month after surgery.

Echocardiographic left ventricular mass LVM (-12%, -23%, -28% and -43% at 1,6,12 and 48 months, respectively) and epicardial fat decreased overtime after sleeve gastrectomy (SG) in humans. In rats with diet-induced obesity, SG significantly reduced LVM and epicardial fat and enhanced the ejection fraction compared with sham-operation. Metabolomics showed a progressive decline of plasma branched-chain amino acids (BCAA), alanine, lactate, 3-OH-butyrate, acetoacetate, creatine and creatinine levels in both humans and rodents.

Hearts of SG rats had a net improvement of BCAA, glucose and fatty acid metabolism and insulin signaling as compared with sham-operated rodents. Supplementation of cardiomyocyte culture medium with BCAAs stimulated the transcription of lipogenic genes and reduced mRNA levels of key mitochondrial β-oxidation enzymes promoting lipid droplet accumulation while glycolysis was enhanced.

**Implications of all the available evidence**

Our results demonstrate that after SG a prompt and sustained decrease of the LVM, epicardial fat, and insulin resistance is seen. Animal and *in vitro* studies suggest a net improvement of cardiac BCAA metabolism together with the amelioration of fat oxidation and insulin signaling and help understanding how SG reduces intra-myocytic fat accumulation and lipotoxicity.

Alt-text: Unlabelled box

## Introduction

The prevalence of severe obesity (i.e., Body Mass Index, BMI ≥40 kg/m^2^) is progressively rising by exponential growth, reaching at present pandemic proportions. According to the World Health Organization (WHO), by 2014 an estimated 39% of the adult global population was with overweight and 13% with frank obesity, doubling its incidence since 1980.^1^ Evolution of modern society has led to an increased consumption of energy-dense foods in conjunction with physical inactivity due to a sedentary lifestyle, causing an energy imbalance and ultimately resulting in overweight and obesity.

Obesity, which is generally associated with left ventricular (LV) hypertrophy,^2^ is a primary risk factor for heart failure, both in men and in women.^3^ Furthermore, obesity and, more specifically, body fat distribution, are relevant in the process of myocardial remodelling. In fact, dysfunctional adipose tissue is preferentially stored in visceral adipose tissue (VAT). Epicardial fat is considered to be the heart-specific VAT, provided with overlapping properties and derivation. Epicardial fat is a highly metabolically active tissue, which has been suggested to exert protective functions over the cardio-metabolic system. However, under pathological states, namely obesity and insulin resistance, its ability to balance the production of anti- and pro-inflammatory adipokines and bioactive molecules is altered, inducing impairment of endothelial function and detrimental changes on myocardial structure and activity.^4^^,^^5^

Several studies have shown the beneficial effects of weight loss on cardiac function and remodelling as assessed by echocardiography, although there is a paucity of literature data regarding prospectively evaluated long-term results.^6^ Interestingly, it has been demonstrated how a 6-month low calorie diet, inducing a weight loss of 7,8%, reduces the left ventricular mass (LVM) by an average of about 5 g independently of dietary composition.^7^

Bariatric surgery currently represents the sole modality treatment deemed to be effective in generating a substantial and durable weight loss. Moreover, surgery produces significant amelioration or complete resolution of obesity-attributable conditions (i.e. type 2 diabetes, dyslipidaemia, hypertension, etc.) allowing it to be now accepted and used as a ‘metabolic surgery’ rather than for mere body weight loss motives.^8^, ^9^, ^10^, ^11^, ^12^, ^13^, ^14^, ^15^ Bariatric-metabolic surgery (BMS) has been additionally proven to reduce long-term cardiovascular events, related morbidity and mortality and its deriving economic encumbrance.^16^, ^17^, ^18^

We hypothesized that sleeve gastrectomy (SG) might determine a prompt and sustained reduction of LVM associated with the improvement of myocardial energy metabolism. To this extent, we have studied the cardiac function by colour Doppler/tissue Doppler imaging echocardiography before and at 1, 6, 12 and 48 months after SG in humans and at 1 month after SG or sham operation in high-fat diet (HFD) induced obesity in rats. Plasma metabolomics in both humans and rodents as well as gene expression of enzymes catabolizing Branched-Chain Amino Acids (BCAA), cell membrane fatty acid transporters, mitochondrial fatty acid transporter and OXPHOS pathway as well as rate-limiting enzymes of glucose metabolism in rat myocardium were studied to understand the metabolic modifications following SG. Since we observed that BCAA circulating levels dramatically decreased early after SG, we sought to elucidate their role on facilitating lipotoxicity and development of insulin resistance in cardiomyocytes *in vitro*.

## Methods

### Human study

#### Outcomes

The primary outcome was the change in LVM from baseline to 12 months. Secondary outcomes included the LVM changes as well as the changes of other echocardiographic parameters, insulin resistance (HOMA-IR) and metabolites at 1, 6, 12 and 48 months after surgery. A secondary goal was to identify the major determinant of LVM reduction after SG.

#### Sample size calculation

A total sample size of 17 subjects was estimated to be required in order to provide 90% power to detect a within-group difference in LVM of 40 g from baseline to 12 months at a 2-sided α of 0.05, assuming a standard deviation of 50. Assuming a retention rate of 80%, a total of 20 subjects were enrolled.

#### Study population

Patients were selected according to the 1991 NIH Consensus Conference weight criteria for bariatric surgery. Therefore, the patients included in our study can be considered representative of the general population affected by morbid obesity undergoing bariatric/metabolic surgery. Twenty patients (9 men and 11 women) aging 45.2 ± 8.5 years, with a BMI of 43.6 ± 7.4 kg/m², with type 2 diabetes (*n* = 10) or with normal glucose tolerance (*n* = 10), were enrolled at the Sapienza University Hospital in Rome, Italy, in this monocentric, prospective study.

Between March 2012 and January 2013, 20 consecutive patients underwent SG. Complete preoperative evaluation was performed by a multidisciplinary team and comprised physical examination, demographics, standard blood chemistry panel, 12-lead electrocardiography and colour Doppler/tissue Doppler imaging echocardiography. The preoperative workup included psychological, nutritional, endocrinological, anesthesiologic, and cardiac evaluations.

Patient inclusion criteria comprised those enlisted by international guidelines.^19^ Exclusion criteria were breastfeeding or pregnancy, a history of acute/chronic coronary syndrome, heart failure with reduced or preserved ejection fraction or cerebrovascular disease. Significant arrhythmias, congenital heart diseases, severe valvular disease were also considered as exclusion criteria.

Patients were studied preoperatively and at 1, 6, 12 and 48 months after SG. Postoperative evaluation included at all time points, clinical assessment, lipid profile changes, systolic and diastolic blood pressures, 12-lead electrocardiography, 2D Doppler/TDI echocardiography, comorbidity progression/remission rates and use of all medications were recorded. T2DM remission was defined as fasting glucose levels >129 mg/dl and haemoglobin A1c (HbA1c) <6.5% without the use of oral and/or injectable hypoglycaemic drugs or history of diabetes and anti-hyperglycaemic drug use. A metabolomic analysis was also performed at 1, 6 and 12 months postoperatively. The 10-year cardiovascular risk was evaluated by means of the Framingham Risk Score.^20^

#### Anthropometry

Body weight was measured to the nearest 0.1 kg with a beam scale and height to the nearest 0.5 cm using a stadiometer (Holatin, Crosswell, Wales, U.K.).

#### Surgical procedure

SG has become amongst the most commonly performed and well-established bariatric procedures, accounting for an estimated 53.6% in 2016, of all bariatric operations worldwide.^21^ Even though initially conceived as the first step of a biliopancreatic diversion with duodenal switch, it is now recognized and accepted as a distinct, stand-alone procedure in view of its effectiveness and safety profile.

SG consists of a longitudinal resection of the stomach along its greater curvature with the use of a 48 F bougie. The ghrelin-rich fundus is completely excised together with part of the gastric body and antrum, with the partial preservation of the antrum and the pylorus. It results in a vertical gastric “sleeve” with a capacity of approximately 60–100 ml.

#### Echocardiography

Echocardiographic follow-up studies were performed as previously reported,^7^ in left lateral position by an experienced echocardiologist (EC), blinded from patient records, using commercially available ultrasonography equipment (CX-50; Philips, Andover, MA, USA) with a 1–5 MHz phased array transducer (S5-1; Philips). The images were acquired from parasternal and apical windows and were digitally recorded according to the American Society of Echocardiography (ASE) standards for offline analysis. M-mode, bidimensional echocardiographic data, pulsed Doppler, continuous and colour Doppler, tissue Doppler Imaging (TDI) flow mapping were recorded. Two expert cardiologists, blinded from the patients’ clinical records, independently reviewed each study and measured the referred quantitative parameter in three consecutive cycles. The parameters of LV geometry were measured offline in M-mode, including the interventricular septum (IVS), posterior wall thickness, and LV internal diameters, measured at the end of diastole (end-diastolic diameter) and at the end of systole (end systolic diameter). When M-mode measurements could not be obtained optimally in three patients (19%), LV internal dimensions and wall thickness measurements were made using the leading edge convention, as described by the ASE.^22^ LV end-diastolic and end-systolic volumes were calculated using the Teichholz method.^22^ Relative wall thickness was calculated as follows:

RWT =2 x LVPW/LVEDD

where RWT is the relative wall thickness, LVPW is the left ventricular posterior wall thickness at end-diastole, and LVEDD is the left ventricular mid-cavity dimension at end diastole.

LVM was calculated using the corrected AES convention^22^ proposed by Devereux:

LVmass (g) = 0.8 × 1.04 × ((LVEDD + LVPWd + IVSd)^3^−LVEDD^3^) + 0.6

For more appropriate normalization of LVM in obese patients, the LVM indexed by height was calculated by dividing the LVM by height in meters raised to the 2.7th power.^23^ Indexation of LVM for the allometric power of its relation to body height (height^2.7^) facilitates identification of LV hypertrophy among obese subjects because height^2.7^ parallels ideal body size for body height, in contrast to actual body weight.^23^ Different methods of indexation of LVM, as for body surface area (BSA), resulted in considerably different prevalence of LV hypertrophy, which is why indexation for body mass index (BMI) or BSA was not considered. LV geometry was considered normal if the relative wall thickness (RWT) was <0.45 and the LVM index (LVMI) was<51 g/m^2.7^. A normal LVMI with increased RWT (>0.45) was considered concentric remodelling. A hypertrophic LV (LVMI >51 g/m^2.7^) was considered eccentric when the RWT was <0.45 and concentric when the RWT was >0.45. Using pulsed-wave Doppler and TDI, early and late diastolic mitral inflow and early diastolic mitral annular velocity, obtained from the average of measurements of the septal and lateral annulus, were recorded to assess diastolic function. The patients were accordingly classified into three groups: normal filling, altered relaxation and pseudonormal filling dynamics. Inter-observer variability was 2.2% for LVEDD, 3.9% for LVESD, 5.1% for LAD, 6.5% for IVS and 3.1% for LVPW. Intra-observer variability was 1.69% for LVEDD, 2.1% for LVESD, 1.42% for LAD, 3.3% for IVS and 3.2% for LVPW. Importantly, both the inter- and intra-observer variability were well below 10%. Inter- and intra-observer variability was assessed by calculating the coefficient of variation, when the same or two different operators performed cardiac ultrasonography, according to the equation

CV=σ/μ•100

Where CV = coefficient of Variation; σ is the standard deviation and µ is the mean.

#### Metabolomic study

Samples for metabolomic analysis were sent in dry ice to the Centre for Research of Magnetic Resonance (CERM), University of Florence, Italy. Frozen serum specimens were brought to room temperature and agitated before use. 300 μl sodium phosphate was added to each sample (70 mM Na2HPO4; 20% v/V 2H2O; 0.025% v/v NaN3; 0.8% w/v sodium trimethylsilyl [2, 2, 3, 3-2h 4] propionate (TSP); pH 7.4) was homogenized in centrifuge for 30 s. 450 ΜL of the mixture was then placed into a 4.25 mm nuclear magnetic resonance tube (Bruker BioSpin srl) for analysis. The NMR spectra 1h for all specimens were acquired with a Bruker 600 MHz spectrometer (Bruker BioSpin) using a 600.13 MHz proton frequency Larmor and equipped with cryo-Probe 5 mm CPTCI 1h-13c/31p-2h with *Z* axis gradient coil, an automatic frequency regulator and an automatic sample loader. A BTO 2000 was used for stabilizing temperatures at a level of 0.1 K. Before measurements, samples were placed for at least 3 min in the NMR probehead for temperature rebalancing (310 K). The one-dimensional spectra (1-D) of the plasma samples were acquired using the sequence spin-echo Carr-Purcell-Meiboom-Gill (CPMG; Bruker) to suppress signals from high molecular weight molecules, a sequence of standard pulses (Noesy Presat), diffusion modified sequence with a diffusion time of 120 ms and fast two-dimensional (2-D) J-Resolved experiments. The free-induction decays were multiplied by an exponential function equivalent to a factor of 1.0 Hz line-broadening before applying the Fourier transform. Processed spectra were corrected automatically by phase and by distortion on a calibrated basis (Tmsp peak at 0.00 ppm) using Topspin (version 2.1, Bruker). Each one-dimensional spectrum in the range between 0.02 and 10.00 ppm was segmented into 0.02 ppm buckets before the statistical analyses. The “bucketing” effectively reduces the total number of variables and to compensate for the small flows in the spectrum.

### Animal study

#### Rodent model

Ten Wistar rats of both sexes aged 10 weeks were housed individually in a controlled room at 22 °C with a 12 h day/night cycle (lights on 0700–1900 h). The animals received a purified tripalmitin-based high fat diet (HFD) ad libitum (Rieper AG, Bolzano, Italy) supplying 71% of energy from saturated fat, although corn oil (1.9/100  g diet) was present in order to prevent essential fatty acid deficiency, 20% from carbohydrate comprising corn starch and sucrose (2:1 weight for weight) and 10% from protein to induce cardiac dysfunction. The HFD was continued for 10 weeks before and 4 weeks after the operation. The animals randomly underwent SG or sham operation. Survival rates were 90% after sham operation and 75% after SG. All experimental procedures were approved by the Catholic University of Rome Institutional Animal Care Committee and all methods were performed in accordance with the relevant guidelines and regulations.

#### Sample size

Based on a previous study^24^ reporting the effects of SG on the Left Ventricular Internal Diameter (LVID) during systole we have hypothesized a lower LVID in SG-operated animals (3.9 mm) compared to the sham-operated group (4.4 mm). Using a value of alpha=0.05, a power of 0.80 and a standard deviation of 0.39, we have calculated that a sample size of 5 animals for each group was sufficient to detect significant differences in cardiac function between groups.

### OGTT

The oral glucose tolerance test (OGTT) was performed at sacrifice. Animals were fasted overnight and then received a 50% D-glucose solution (1 g/kg body weight) by oral gavage. Blood was collected from the tail vein to measure glucose and insulin concentrations at 0, 15, 30, 60, 90,120 and 180 min at the end of the study. After centrifugation, plasma was divided into aliquots and stored at -20 ^°^C until analyses.

#### Echocardiography

Rats were anesthetized in a sedation chamber containing 96% O_2_ and 4% isoflurane before mask ventilation with a mixture of 98.25% O_2_ and 1.75% isoflurane. Transthoracic echocardiography was performed using a Vevo 2100 system (FUJIFILM VisualSonics Inc., Canada). End-systolic and end-diastolic dimensions, end-systolic and end-diastolic volumes and stroke volume were recorded in order to calculate the per cent fractional shortening (FS%) and ejection fraction (EF%). Each measure was repeated three times.

#### Lipid staining

Oil Red O was performed to assess intracellular lipid accumulation. Slides were fixed overnight with 4% formaldehyde and stained with Oil Red O solution for 1 h. Counterstain was performed with Haematoxylin solution. Photographs were taken with an optical microscope (ZEISS Primo Star HAL/LED).

Primary adult rat cardiomyocytes were stained with Nile Red (100 ng/mL) for 45 min. Images of lipid droplets were taken using confocal microscope Nikon A1 RHD25 and NIS-Elements imaging software was used to analyse images.

#### Interventions

The rats were anesthetized using ketamine (75  mg/kg intramuscularly) and xylazine (10  mg/kg intramuscularly). Ten millilitres of sterile 0.9% NaCl were administered subcutaneously before surgery. Access to the peritoneal cavity was obtained by a 3 cm laparotomy.

#### Vertical sleeve gastrectomy

A midline laparotomy was performed and the stomach was exposed outside the abdominal cavity and placed on saline-moistened gauze pads. The gastroepiploic and gastro-splenic ligaments were divided along the greater curvature. Approximately 80% of the stomach was excised and subsequently sutured with 5-0 prolene, leaving a tubular gastric remnant in continuity with the oesophagus and with the pylorus and duodenum. After the operation, the abdominal wall was closed in layers.

In sham-operated rats, a midline laparotomy was performed and the stomach was exposed and gently manipulated. The abdominal cavity was kept open for the same amount of time required to perform the other operations. A 1 cm gastrotomy was performed and then closed as in the SG group.

#### Postoperative care

At the end of the surgical procedures, rats received sterile 0.9% NaCl 10  ml i.p. and 10  ml s.c. to preserve hydration throughout healing. The animals received ketoprofen 5  mg/kg as an analgesic, were placed on a heated mat until they recovered and then were replaced into their cages. The rats were allowed to drink purified water for 12  h after surgery, then a liquid diet (5% glucose and 0.2% KCl) was provided for the next 48  h. They were given a HFD 4 weeks postoperatively.

#### Plasma metabolomics

*Samples preparation for ^1^H-NMR spectroscopy*: Frozen plasma samples were thawed at room temperature and shaken before use. A total of 100 µL of a sodium phosphate buffer (70 mM Na_2_HPO_4_; 20% (v/v) ^2^H_2_O; 0.025% (v/v) NaN_3_; 0.8% (w/v) TMSP, pH 7.4) was added to 100 µL of each plasma sample, and the mixture was homogenized by vortexing for 30 s. A total of 180 µL of this mixture was transferred into a 3 mm NMR tube (Bruker BioSpin srl) for the analysis.

*NMR spectra acquisition*: For each plasma sample, one-dimensional ^1^H NMR spectra were acquired using a Bruker 600 MHz spectrometer (Bruker BioSpin) operating at 600.13 MHz proton Larmor frequency and equipped with a 5 mm CPTCI ^1^H-^13^C-^31^P and ^2^H-decoupling cryoprobe including a *z* axis gradient coil, an automatic tuning-matching (ATM) and an automatic sample changer. To stabilize the temperature at the level of approximately 0.1 K at the sample, a BTO 2000 thermocouple was used. Before starting measurements, samples were kept for at least 5 min inside the NMR probehead, for temperature equilibration at 310 K.

According to standard practice, for each plasma samples three one-dimensional ^1^H NMR spectra with different pulse sequences were acquired:

(i) a standard nuclear Overhauser effect spectroscopy pulse sequence NOESY 1Dpresat (noesygppr1d.comp; Bruker BioSpin)^3^, using 64 scans, 98304 data points, a spectral width of 18028 Hz, an acquisition time of 2.7 s, a relaxation delay of 4 s and a mixing time of 0.01 s, was applied to obtain a spectrum in which both signals of low molecular weight metabolites and high molecular weight macromolecules (lipids and lipoproteins) are visible.
(ii) a standard spin echo Carr-Purcell-Meiboom-Gill (CPMG)^4^ (cpmgpr1d.comp; Bruker BioSpin) pulse sequence, with 64 scans, 73728 data points, a spectral width of 12019 Hz and a relaxation delay of 4 s, was used to selectively observe low molecular weight metabolites, suppressing signals arising from high molecular weight aggregates.
(iii) a standard diffusion-edited^5^ (ledbgppr2s1d.comp; Bruker BioSpin) pulse sequence, using 64 scans, 98304 data points, a spectral width of 18028 Hz and a relaxation delay of 4 s, was applied to suppress the signals of low molecular weight metabolites.

*Spectral processing*: Before applying Fourier transformation, free induction decays were multiplied by an exponential function equivalent to a 0.3 Hz line-broadening factor. All transformed spectra were automatically corrected for phase and baseline distortions and calibrated to the anomeric glucose doublet at 5.24 ppm using TopSpin 3.2 (Bruker Biospin srl).

#### Quantitative real-time PCR analysis

Total RNA from heart biopsies was extracted using the RNeasy Fibrous Tissue Mini kit (Qiagen GmbH, Hilden, Germany) according to the indications provided by the company. A small aliquot of the total RNA obtained (1 μL) was subjected to qualitative and quantitative control by using the microdrop (Thermo Fisher Scientific, Waltham, Massachusetts). We qualitatively and quantitatively assessed the individual samples using dedicated software. Total RNA was reverse-transcribed into cDNA by using iScript RT (Bio-Rad, Hercules, California). SYBR Green gene expression assays were performed in triplicate according to the manufacturer's instructions using the iQ SYBR Green Supermix (Bio-Rad, Hercules, California) and the iQ5 Multicolour Real-Time PCR Detection System (Bio-Rad, Hercules, California). The pairs of primers used are listed in Table S1.

#### Western blot analysis

Heart biopsies were homogenized in RIPA buffer containing a cocktail of protease inhibitors. Homogenates were cleared by centrifugation (19.000 g; 30 min, 4 ^°^C). Protein content was determined using Bradford Protein Assay (Bio-Rad Laboratories, Hercules, CA). Protein lysates (30 μg) were separated on 10% SDS-PAGE and transferred on PVDF membrane. Membranes were probed overnight with phospho-AktSer473 (Cell Signaling Technology Cat# 4060, RRID: AB_2315049), phospho- AMPK Thr172 (Cell Signaling Technology Cat# 4188, RRID: AB_2169396), OXPHOS (Abcam Cat# ab110413, RRID:AB_2629281), BCAT2 (Thermo Fisher Scientific Cat# PA5-87314, RRID:AB_2804058), BCKDH (Thermo Fisher Scientific Cat# PA5-76434, RRID:AB_2720161), GAPDH (Santa Cruz Biotechnology Cat# sc-51905, RRID: AB_629535) and Tubulin (Santa Cruz Biotechnology Cat# sc-5286, RRID:AB_628411). Membranes with phospho-antibodies were stripped for 30 min at 56 °C and re-probed overnight with total Akt (Cell Signaling Technology Cat# 9272, RRID: AB_329827) and AMPK (Cell Signaling Technology Cat# 2603, RRID: AB_490795), respectively. Detection and analysis were performed with Chemidoc XRS Image system and Image Lab 5.0 software (Bio-Rad Laboratories, Hercules, CA).^2^^,^^10^ OXPHOS proteins were normalized with Tubulin. BCAT2 and BCKDH were normalized with GAPDH, while phospho-AktSer473 and AMPK Thr172 were normalized with total Akt and AMPK.

#### Adult cardiomyocyte isolation and culture

Primary adult cardiomyocytes were isolated from rats following a standard enzymatic digestion protocol. Briefly, rats were anesthetized and hearts perfused with Ca^2+−^free buffer containing: 120 mM NaCl, 4.7 mM KCl, 10 mM HEPES, 0.6 mM KH_2_PO_4_, 0.6 mM Na_2_HPO_4_, 1.2 mM MgSO_4_, 4.6 mM NaHCO_3_, 30 mM taurine, 10 mM 2,3-Butanedione monoxime, 5.5 mM glucose and 15.000 U collagenase type II, pH 7.4 at 37 ^°^C. The heart was then minced and cardiomyocytes were resuspended in serum-free Medium 199 (Sigma Aldrich, M2520) supplemented with 5% FBS (Thermo Fisher Scientific), 26 mM NaHCO_3_, 10 mM glutathione and antibiotics (100 IU/ml penicillin + 10 mg/ml streptomycin, Thermo Fisher Scientific) and plated on laminin (10 μg/ml, Sigma Aldrich) precoated plates. After an adhesion period of 2 h, cardiomyocytes were cultured in Medium 199 without FBS for 24 h and BCAAs (2.15 mM) or insulin (100 nM) were added.

### Statistics

Continuous variables are summarized as mean and standard errors. Repeated-measurements analysis of variance (ANOVA) was used to assess general differences across four time points (1,6,12 and 48 months after sleeve gastrectomy). Normality of the residuals of the models was evaluated visually and using the Kolmogorov-Smirnov test. For skewed variables, logarithmic transformation was used to achieve normality.

After visually checking for a linear relationship between each two variables scatterplot, Pearson correlation coefficients were calculated to assess the correlations among the variables studied.

Multiple linear regression analysis with centre adjustment was used to establish the independent determinants of the left ventricular mass adjusted by the body surface area.

*P* values for multiple comparisons were adjusted using the step-down Bonferroni method of Holm.

Two-sided *P* values less than 0.05 were considered statistically significant. Statistical analyses were performed using SPSS version 25.

We performed metabolomic statistical analysis using MetaboAnalyst 0.web tool (www.metaboanalyst.ca). We checked for data integrity and missing value using Singular Value Decomposition (SVDIMPUTE) algorithm. Data were log transformed and scaled using mean centering algorithm.

Sparse version of the partial least-squares discriminant analysis (sPLS-DA) was performed to visualize metabolic differences between groups. sPLS-DA imposes sparseness within the latent components of the model in order to promote variable selection and simultaneously performs dimension reduction. sPLS-DA has highly predictive performances and is well able to select informative variables to predict sample clustering.^25^

The predictability of the model was determined by internal validation using the ten-fold cross-validation of R2, Q2 value and permutation test.

#### The best-fitted PLS-DA model was used to explore the variable importance in the projection (VIP) score of all metabolites. Potential differential metabolites were validated by Student's *t*-test (*P* value<0.05) and VIP score (>1)

The statistical analysis used was a combination of the PLS-DA and Canonical Correlation analysis (CA), followed by the Support Vector Machines (SVM) performed on the CA score.

The heat-map was used as a graphical representation of metabolomic average values per experimental categories including only metabolites for which the procedure interaction from the mixed-effect analyses was significant. To identify the metabolic pathways involved, the validated metabolites were annotated with Human Metabolome Database (http://www.hmdb.ca/). Materials supporting the results or analyses presented in this paper is deposited in the online repository Figshare (doi: 10.6084/m9.figshare.18550760).

#### Ethics

The study was reviewed and approved by the institutional human ethics committee of the University Sapienza in Rome, Italy (Reference number: 127/18 RIF.CE4915), in accordance with the national guidelines and the provisions of the Helsinki Declaration, as revised in 2000. All participants provided written informed consent to participate in the study. Additional written informed consent was obtained prior to any surgical procedure. The animal study was performed in compliance with animal use guidelines and ethical approval was also obtained (Reference number: IF295.49).

#### Role of funding source

This study was funded by internal funds from University of Rome Sapienza which had a role in performing metabolomics analyses. The funders had no role in the study design, interpretation or writing of the report.

## Results

### Human data

#### SG induces a prompt, durable weight loss and a net improvement of insulin resistance

Table 1 reports weight, plasma glucose, lipid profile and blood pressure over time. At 1-year follow-up, body weight was reduced by 36% and weight loss maintained until the end of the study (-33%, (*P* < 0.0001 Holm-Bonferroni method), despite an approximate 10% weight regain by 48 months postoperatively.

**Table 1:** Anthropometric and metabolic data of patients at baseline and follow-up. Significance was assessed using repeated-measurements analysis of variance (ANOVA). Data are shown as mean±SE.

Follow-up after Sleeve Gastrectomy					
	Baseline	Month 1	Month 6	Month 12	Month 48
Weight (kg)	123.8 ± 22.8	110.6 ± 19.6	85.9 ± 15.9	78.8 ± 14.8	82.8 ± 19.9
*P*		0.335	<0.0001	<0.0001	<0.0001
BMI (kg/m^2^)	43.6 ± 7.4	38.8 ± 6.4	30.1 ± 5.0	27.9 ± 4.7	28.9 ± 7.4
*P*		0.172	<0.0001	<0.0001	<0.0001
Total cholesterol (mmol/l)	5.1 ± 0.9	4.6 ± 1.0	4.8 ± 0.7	5.1 ± 0.9	4.9 ± 0.4
*P*		1	1	1	1
HDL-cholesterol (mmol/l)	1.2 ± 0.4	1.2 ± 0.4	1.3 ± 0.3	1.6 ± 0.4	1.7 ± 0.4
*P*		1	1	0.038	0.004
LDL-cholesterol (mmol/l)	3.4 ± 0.9	2.7 ± 0.9	2.9 ± 0.8	2.9 ± 1.1	2.7 ± 0.3
*P*		0.224	1	1	0.727
Triglycerides (mg/dl)	2.0 ± 0.9	1.5 ± 0.5	1.3 ± 0.5	1.0 ± 0.4	1.0 ± 0.5
*P*		0.052	0.005	<0.0001	0.003
Glucose (mmol/l)	6.9 ± 1.8	5.8 ± 1.4	5.3 ± 1.2	5.1 ± 1.7	5.4 ± 1.1
*P*		0.161	0.007	0.004	0.128
Insulin (pmol/l)	109.1 ± 20.2	67.3 ± 9.1	62.2 ± 11.9	42.9 ± 5.2	42.1 ± 11.4
*P*		0.194	0.089	0.004	0.011
HOMA-IR	6.4 ± 1.5	3.0 ± 0.5	2.5 ± 0.5	1.6 ± 0.3	1.8 ± 0.6
*P*		0.022	0.004	<0.0001	0.003
Systolic Blood Pressure (mmHg)	140.8 ± 11.4	123.8 ± 12.5	119.2 ± 10.6	116.5 ± 8.6	122.5 ± 15.2
*P*		<0.0001	<0.0001	<0.0001	<0.0001
Diastolic Blood Pressure (mmHg)	88.3 ± 6.5	76.8 ± 9.5	72.9 ± 8.9	70.9 ± 10.3	79.6 ± 9.9
*P*		0.001	<0.0001	<0.0001	0.1
					

Insulin resistance decreased by more than 50% just 1 month after SG and plateaued at more than 70% reduction at 48 months following surgery (Table 1).

Total- and LDL-cholesterol did not change significantly, while HDL-cholesterol significantly (*P* = 0.004 Holm-Bonferroni method) increased at 48 months. Triglycerides decreased from 179.16 ± 18.13 to 102.90 ± 4.96 mg/dl at 48 months (*P* = 0.003 Holm-Bonferroni method).

Systolic blood pressure decreased from 140.75 ± 2.55 to 122.50 ± 4.37 (*P* < 0.0001 Holm-Bonferroni method) and diastolic blood pressure was reduced from 88.25 ± 1.46 to 69.58 ± 2.85 mmHg (*P* < 0.0001 Holm-Bonferroni method) at the end of the study.

The use of cardiovascular and glucose-lowering medications, including insulin, was drastically reduced already at the first month following surgery and continued lowering with time (Table S2).

#### SG is associated with a net reduction of LVM and epicardial fat thickness

Increased fat-free mass and fat mass in obesity drives a higher cardiac output and workload, and consequently promotes LVM rise and heart remodelling^26^^,^^27^ in concomitance with lack of adjustment of peripheral resistance.^28^

The left ventricular mass decreased by 12% at 1 month after SG and further declined at 6 months (−23%) and at 12 months (−28%) after surgery. At 48 months following SG the reduction of LVM was −43% (Table 2).

**Table 2:** Changes in cardiac parameters after Sleeve Gastrectomy.

Echocardiographic Variables	Baseline	Follow-up after Sleeve Gastrectomy			
		Month 1	Month 6	Month 12	Month 48
Cardiac Output (l/min)	6.5 ± 1.5	5.8 ± 1.1	5.7 ± 1.2	5.4 ± 1.2	6.0 ± 1.6
*P*		1	0.784	0.201	1
End Diastolic Diameter (mm)	53.3 ± 4.6	51.9 ± 4.3	50.8 ± 3.7	51.1 ± 3.3	49.5 ± 3.8
*P*		1	0.646	1	0.113
End Systolic Diameter (mm)	33.7 ± 5.0	32.6 ± 3.6	31.8 ± 3.6	31.8 ± 3.3	30.8 ± 3.4
*P*		1	1	1	0.436
Interventricular Septum (mm)	12.2 ± 1.9	11.4 ± 1.6	10.8 ± 1.5	9.7 ± 1.5	8.9 ± 1.3
*P*		1	0.087	<0.0001	<0.0001
Posterior Wall (mm)	11.1 ± 1.6	10.9 ± 2.4	9.8 ± 0.8	9.1 ± 1.5	8.1 ± 1.2
*P*		1	0.108	0.004	<0.0001
Left Ventricular Mass (g)	253.0 ± 70.2	222.8 ± 56.3	195.8 ± 44.4	181.8 ± 49.9	145.1 ± 41.8
*P*		0.852	0.019	0.002	<0.0001
Relative Wall Thickness (ratio)	0.4 ± 0.1	0.4 ± 0.1	0.4 ± 0.1	0.4 ± 0.1	0.3 ± 0.04
*P*		1	1	0.006	<0.0001
Ejection Fraction (%)	61.7 ± 4.6	63.2 ± 3.0	63.3 ± 3.7	65.5 ± 3.6	64.8 ± 3.3
*P*		1	1	0.029	0.271
Aortic Root Diameter in Diastole (mm)	30.8 ± 3.1	30.8 ± 2.9	30.5 ± 3.0	30.1 ± 2.9	30.8 ± 3.4
*P*		1	1	1	1
Aortic Root Diameter in Systole (mm)	31.6 ± 2.9	31.8 ± 2.9	31.8 ± 2.9	31.7 ± 2.8	32.2 ± 3.3
*P*		1	1	1	1
Aortic Root Distensibility (cm^2^·dyn^− 1^·10^− 6^)	1.1 ± 0.7	1.5 ± 0.7	1.9 ± 1.01	2.3 ± 0.8	2.4 ± 1.3
P		1	0.042	0.001	0.002
Aortic Pressure Strain (%)	2.7 ± 1.3	3.5 ± 1.5	4.4 ± 2.0	5.2 ± 1.6	4.7 ± 1.7
*P*		1	0.026	<0.0001	0.014
Aortic Stiffness Index (ratio)	1.2 ± 0.5	1.7 ± 0.8	2.1 ± 0.8	2.6 ± 0.9	1.9 ± 0.7
					
*P*		0.762	0.005	<0.0001	0.078
Aortic Pressure Strain Modulus (cm^2^·dyn^− 1^·10^− 6^)	2.5 ± 1.6	1.8 ± 0.9	1.2 ± 0.6	0.9 ± 0.4	1.1 ± 0.6
*P*		0.204	0.001	<0.0001	0.001
Left Atrium Diameter (mm)	39.0 ± 3.9	37.4 ± 3.9	35.7 ± 3.7	36.1 ± 2.8	37.2 ± 3.9
*P*		1	0.07	0.166	1
Left Atrium Area (cm^2^)	20.8 ± 3.2	19.4 ± 2.9	18.3 ± 1.9	19.1 ± 2.8	19.3 ± 2.4
*P*		1	0.073	0.672	1
Iso-Volumetric Contraction Time (msec)	70.8 ± 16.7	66.7 ± 15.2	64.4 ± 17.8	68.5 ± 18.5	74.4 ± 14.4
*P*		1	1	1	1
Iso-Volumetric Relaxation Time (msec)	132.5 ± 24.9	121.9 ± 16.9	114.8 ± 16.9	98.9 ± 19.6	111.9 ± 14.7
*P*		0.855	0.06	<0.0001	0.046
Ejection Time (msec)	264.5 ± 21.9	272.2 ± 25.2	294.5 ± 29.0	294.9 ± 27.6	287.9 ± 33.6
*P*		1	0.01	0.01	0.201
Myocardial *P* erformance Index (ratio)	0.8 ± 0.2	0.7 ± 0.1	0.6 ± 0.1	0.6 ± 0.1	0.7 ± 0.1
*P*		0.301	<0.0001	<0.0001	0.061
E/E'l (ratio)	7.1 ± 2.1	6.4 ± 1.6	7.5 ± 2.4	7.2 ± 2.3	6.1 ± 1.2
*P*		1	1	1	1
Right Ventricular Diameter (mm)	31.3 ± 3.2	30.1 ± 2.9	29.5 ± 2.9	28.5 ± 4.4	27.7 ± 3.1
*P*		1	1	0.132	0.045
Tricuspid Annulus S' wave velocity (cm/sec)	13.5 ± 2.2	12.4 ± 1.6	12.4 ± 1.9	12.4 ± 1.7	13.7 ± 1.8
*P*		0.88	0.798	0.94	1
Systolic Pulmonary Artery Pressure (m/sec)	25.9 ± 3.9	27.2 ± 3.1	26.2 ± 3.1	23.5 ± 3.6	23.4 ± 3.0
*P*		1	1	0.555	0.633
Epicardial Fat Thickness (mm)	10.7 ± 1.8	9.8 ± 1.5	8.9 ± 1.7	8.1 ± 1.9	5.4 ± 1.4
*P*		0.987	0.022	<0.0001	<0.0001

Epicardial fat thickness constantly decreased over time and halved at 48 months after SG (Supplementary Figure 1) (Table 2) becoming similar to the values observed in normal weight subjects.^29^

In a multiple regression analysis (R^2^ = 0.64, *P* < 0.0001 Holm-Bonferroni method), the best predictors of LVM reduction were weight (*P* < 0.0001 Holm-Bonferroni method; part correlation=0.28), metabolomics creatinine (*P* < 0.0001 Holm-Bonferroni method; part correlation=0.30), epicardial fat thickness (*P* = 0.001 Holm-Bonferroni method; part correlation=0.25) and systolic blood pressure (*P* = 0.008 Holm-Bonferroni method; part correlation=0.20).

#### Metabolomics: after SG branched-chain amino acids decrease, lactate and ketone bodies over time

**Figure 1:**
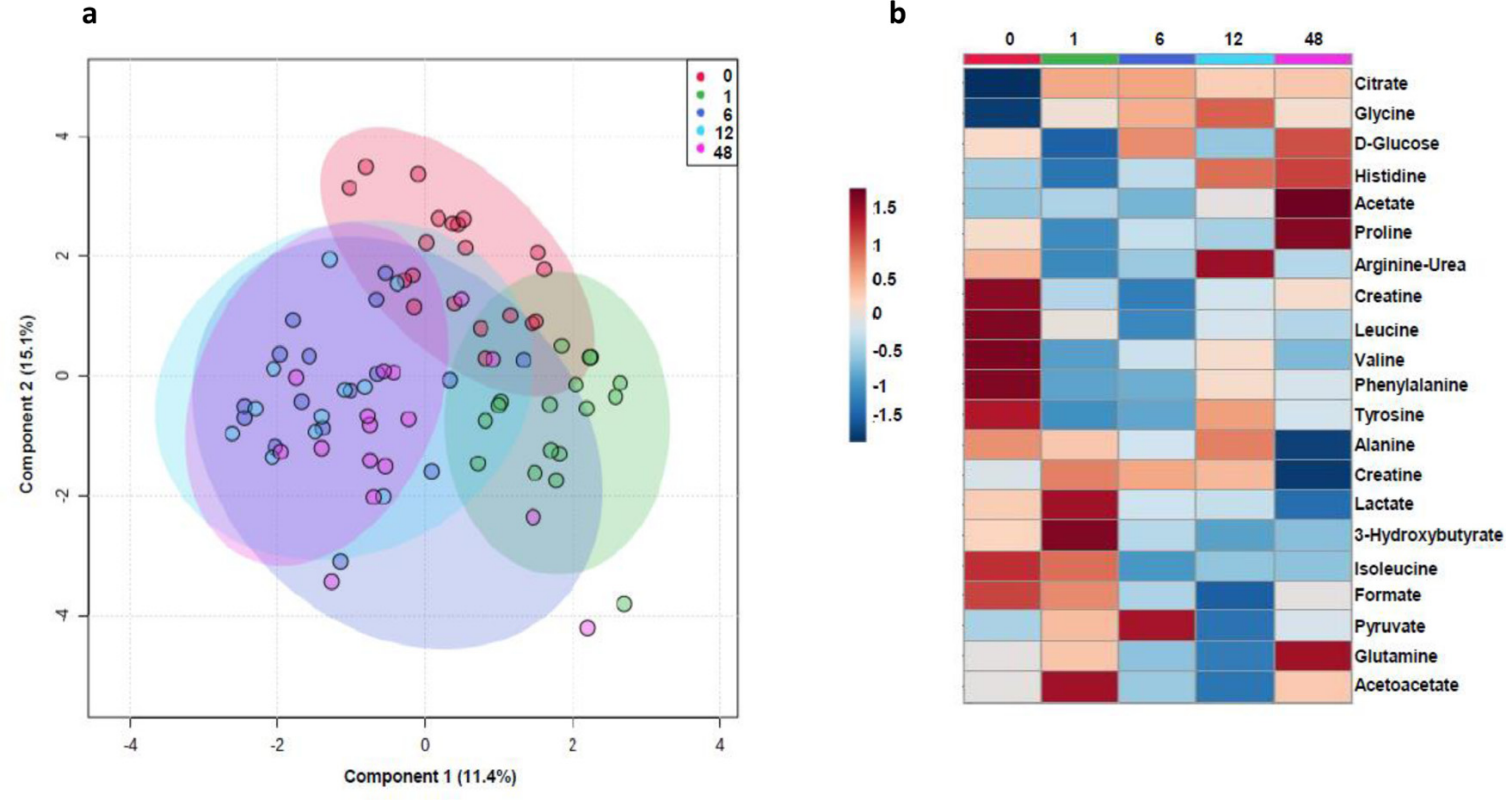
Human metabolomics analysis. **Panel a:** sPLS-DA explains 26.5% of the variance of the variables of interest. **Panel b:** Heat map of metabolites before and 1, 6, 12 and 48 months after SG. sPLS-DA, Partial least-squares discriminant analysis. (Sample size *n* = 20)

As shown in Figure 1 Panel a, sPLS-DA explains 26.5% of the variance of the variables of interest, however, while baseline data and data at 1 month following SG are well separated, data at 6, 12 and 48 months are somewhat overlapping suggesting a certain stability in the modification of metabolite patterns after SG.

Figure 1 Panel b shows the heat map revealing that BCAA valine, leucine and isoleucine as well as alanine, lactate, 3-OH-butyrate, acetoacetate, creatine and creatinine decrease over time.

After SG, 43% of the metabolites changed significantly, but only four of them had a VIP score >1 (Supplementary Figure 2, Panel a).

Supplementary Figure 2, Panel b, shows the metabolites that correlate with lactate; those with the highest correlations are pyruvate, alanine and 3-OH-butyrate.

### Rodent data

#### SG reduces body weight and improves insulin sensitivity

**Figure 2:**
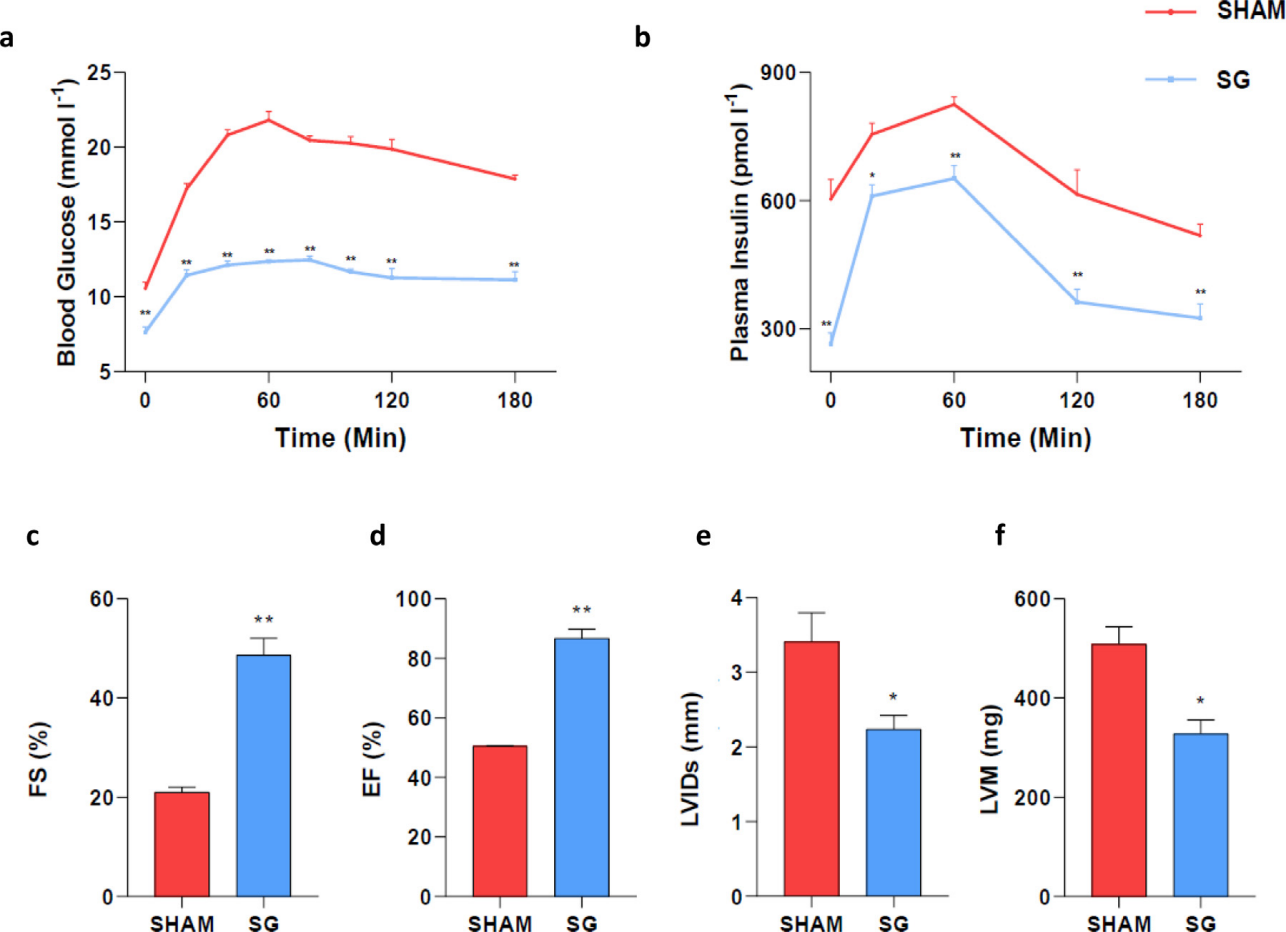
SG impact on insulin sensitivity and cardiac echocardiographic parameters. **Panels a,b:** Time courses of blood glucose and plasma insulin after the OGTT. **Panels c,d:** Fractional shortening (c) and ejection fraction (d) were significantly increased in SG group. **Panels e,f:** LV internal diameter during systole and LV mass were significantly decreased in SG group. OGTT, Oral Glucose Test Tolerance; FS, Fractional shortening; EF, Ejection fraction; LVID, Left Ventricular internal diameter during systole; LVM, Left Ventricular mass. ^⁎⁎^*P* = 0.008; *P = 0.016 (Holm-Bonferroni method). Data are mean ± SEM (*n* = 5 rat per group).

At the end of the study body weight was 40% lower in rats undergone SG than in sham-operated rats (336.40 ± 10.33 vs. 563.01 ± 35.06 g, *P* = 0.002 Holm-Bonferroni method). The time courses of blood glucose and plasma insulin after the OGTT are depicted in Figure 2, Panels a and b, respectively. Both blood glucose (*P* < 0.0001 Holm-Bonferroni method) and plasma insulin AUCs (*P* = 0.001 Holm-Bonferroni method) were significantly reduced in the SG group as compared with the sham group. Therefore, the glucose AUC to insulin AUC ratio was significantly lower in SG than in sham-operated rats (0.024 ± 0.0008 vs. 0.028 ± 0.001; *P* = 0.032 Holm-Bonferroni method) showing that a smaller amount of insulin was required to clear circulating glucose. HOMA-IR, which is a surrogate of hepatic insulin resistance, was significantly lower in SG than in sham-operated animals (13.40 ± 0.57 and 39.55 ± 3.24, respectively; *P* = 0.0079 Holm-Bonferroni method), showing a much better hepatic insulin sensitivity following SG.

#### SG reduces LVM and epicardial fat thickness

Echocardiograms, performed 4 weeks after surgery, showed a significant improvement of the systolic function in SG-operated animals compared with sham-operated rats. Systolic function, assessed by fractional shortening (46.68 ± 3.32 vs. 21.06 ± 0.99 %; *P* = 0.008 Holm-Bonferroni method) and ejection fraction (86.75 ± 3.07 vs. 50.53 ± 0.20 %; *P* = 0.008 Holm-Bonferroni method), were significantly improved after SG compared with the sham operated group (Figure 2, Panels c and d). The increased LV function in the SG group was associated with a significant decrease of LV internal diameter during systole (LVID) (2.24 ± 0.19 vs. 3.41 ± 0.39 mm; *P* = 0.016 Holm-Bonferroni method) and LVM (328.3 ± 27.50 vs. 508.6 ± 34.98 mg; =0.016 Holm-Bonferroni method) (Figure 2, Panels e and f).

Echocardiographic parameters show a net improvement in systolic and LV function following SG.

#### Metabolomics: SG reduces circulating levels of lactate and branched chain amino acids

**Figure 3:**
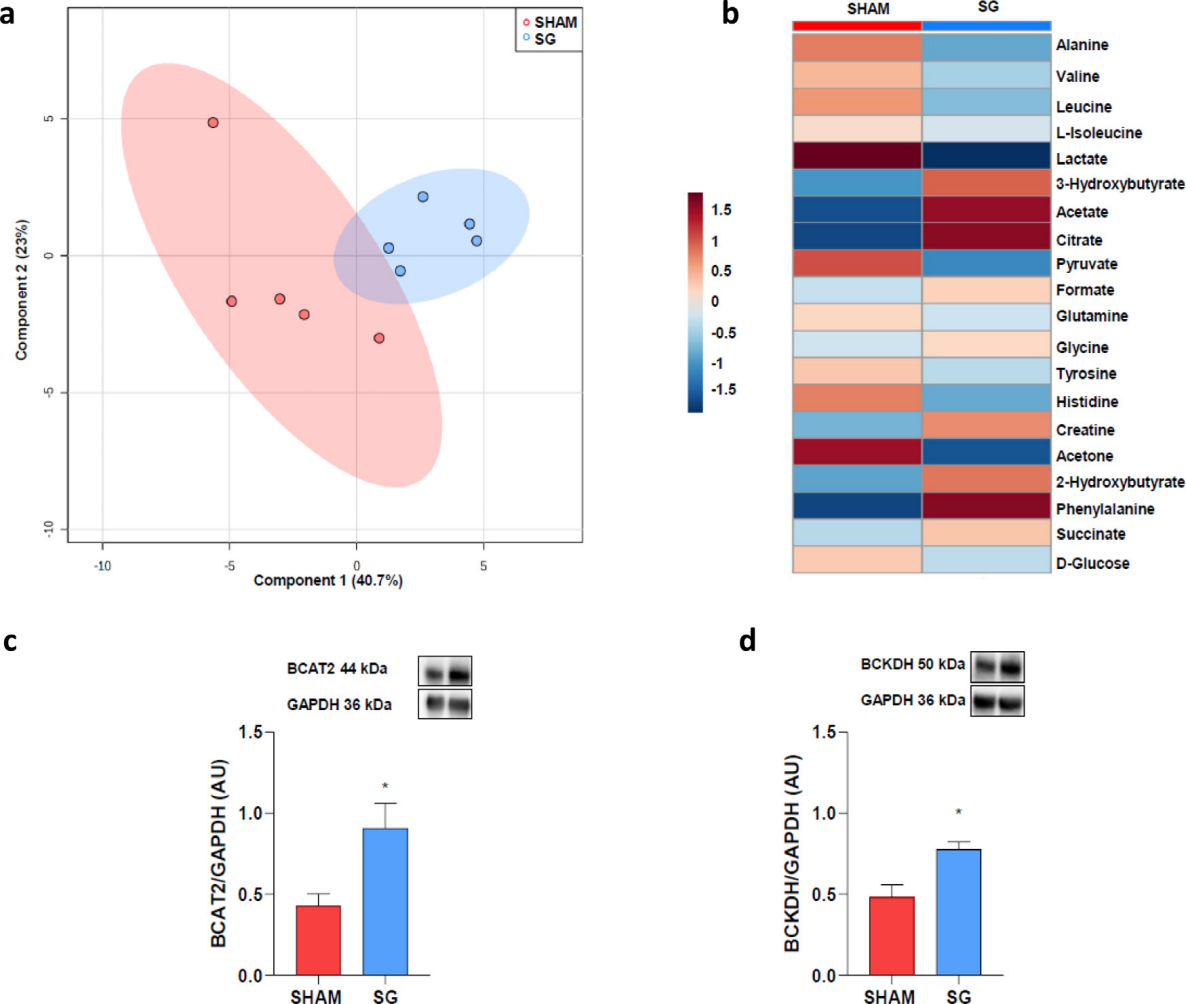
Rodents metabolomics analysis and BCAA metabolism. **Panel a:** sPLS-DA explains 63.7% of the variance of the variables of interest. **Panel b:** Heat map of metabolites before and 1 months after SG. sPLS-DA, Partial least-squares discriminant analysis. **Panels c-d:** BCAT2 and BCKDH protein expression increased in the heart of SG rats when compared to sham-operated animals. Glyceraldehyde 3-phosphate dehydrogenase (GPDH). BCAT2, Branched Chain Amino Acid Transaminase 2; BCKDH, branched-chain α-ketoacid dehydrogenase complex. *P = 0.016 (Holm-Bonferroni method). Data are mean ± SEM (*n* = 5 rat per group).

The plasma metabolic profile was assessed in the SG and sham groups. After SG, 25% of the metabolites changed significantly and nine had a VIP score >1 (Supplementary Figure 3). The first two components of the PLS-DA explained 63.7% of the variance of these metabolites (Figure 3, Panel a). The heat map (Figure 3, Panel b) shows that BCAA and lactate were lower, while 3-hydroxybutyrate was higher after SG than after sham-operation.

In line with the plasma metabolomic results, we observed that protein levels of Branched Chain Amino Acid Transaminase 2 (BCAT2) and branched-chain keto-acid dehydrogenase (BCKDH) - two enzymes that tightly regulate BCAA catabolism - were significantly higher in rats that underwent SG than in sham-operated animals (Figure 3 Panels c and d).

Plasma metabolomic reveals a shift towards metabolites used by cells to produce ATP following SG, suggesting a cardiac metabolic reprogramming with increased energy production.

#### SG increases the expression of genes involved in branched chain amino acid metabolism, fatty acid transportation, OXPHOS and as well as insulin signalling in rat myocardium and reduces myocardial fat accumulation

We investigated the expression of proteins implicated in BCAA metabolism, oxidative phosphorylation (OXPHOS, protein complexes I–V) and insulin signalling (Akt Ser473 and AMPK Thr172), as well as the mRNA expression of representative genes involved in fatty acid (CD36, FABP3, FATP1, CPT-1 and PPARα) and glucose metabolism (GLUT1, GLUT4 HK, PK and LDH) in myocardium of rats undergoing SG or sham-operation.

We found that glucose transportation was enhanced and glucose and lipid oxidation increased in the heart of rodents following SG as compared with sham-operation.

**Figure 4:**
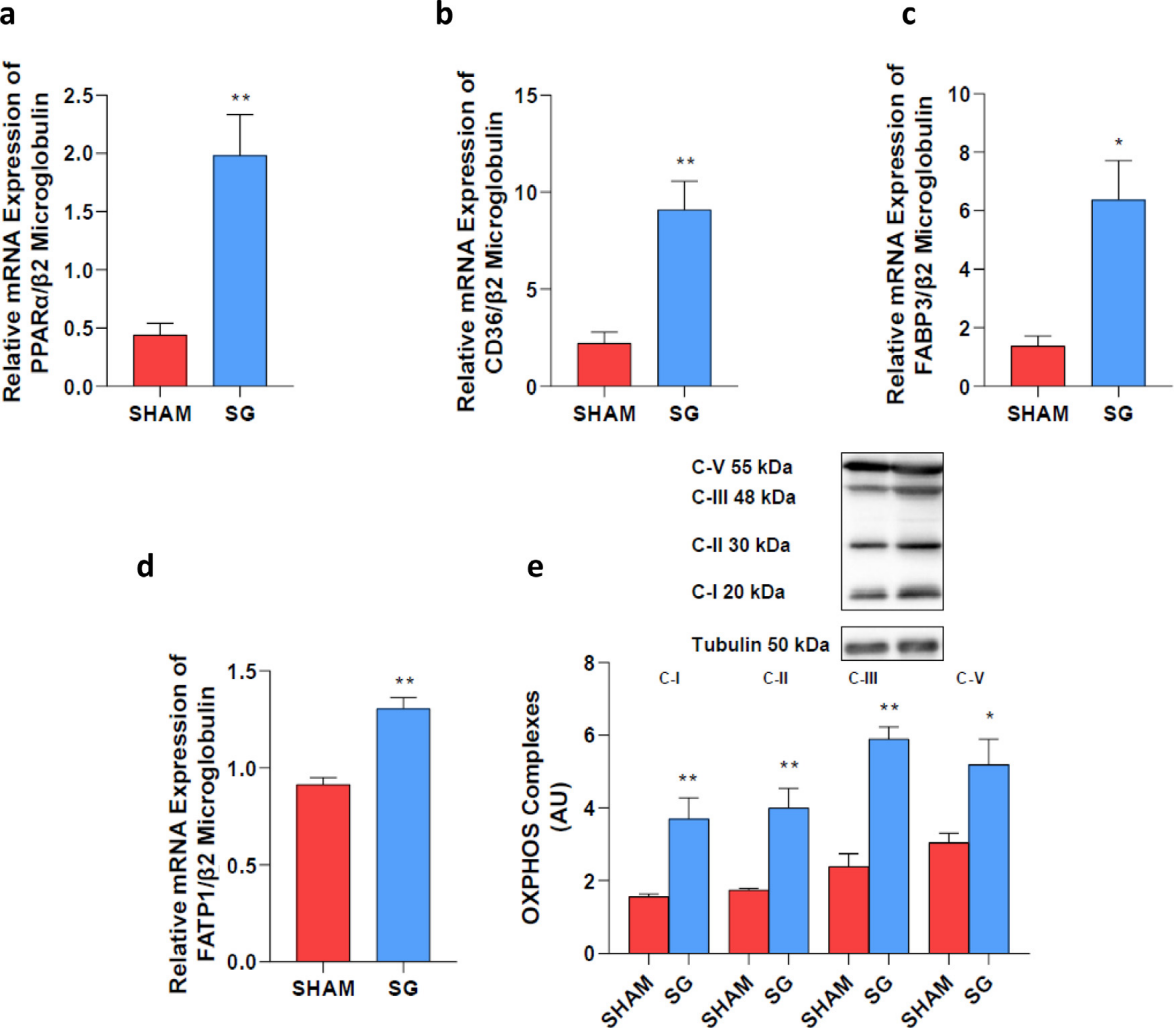
SG increases the expression of genes involved in fatty acid transportation and OXPHOS. **Panel a:** PPARα mRNA expression was significantly higher in SG than in sham-operated rats. **Panels b–d:** Fatty acid transporters mRNA expression was significantly increased after SG compared to sham-operated rats. **Panel e:** OXPHOS proteins were upregulated in SG compared with sham-operated rats. PPARα, Peroxisome proliferator-activated receptor α; CD36, Cluster of differentiation 36; FABP, Fatty Acid-Binding Protein; FATP1, fatty acid transport protein 1; C-I/II/III/V, Complex I/II/III/V. ^⁎⁎^*P* < 0.008; *P < 0.032 (Holm-Bonferroni method). Data are mean ± SEM (*n* = 5 rat per group).

The nuclear receptor peroxisome proliferator activated receptor alpha (PPARα), which is considered a master switch for heart metabolic remodelling, was significantly higher in SG than in sham-operated rats (Figure 4, Panel a).

Fatty acid transporters and OXPHOS genes were upregulated in SG compared with sham rats (Figure 4, Panels b–e; Table S3).

**Figure 5:**
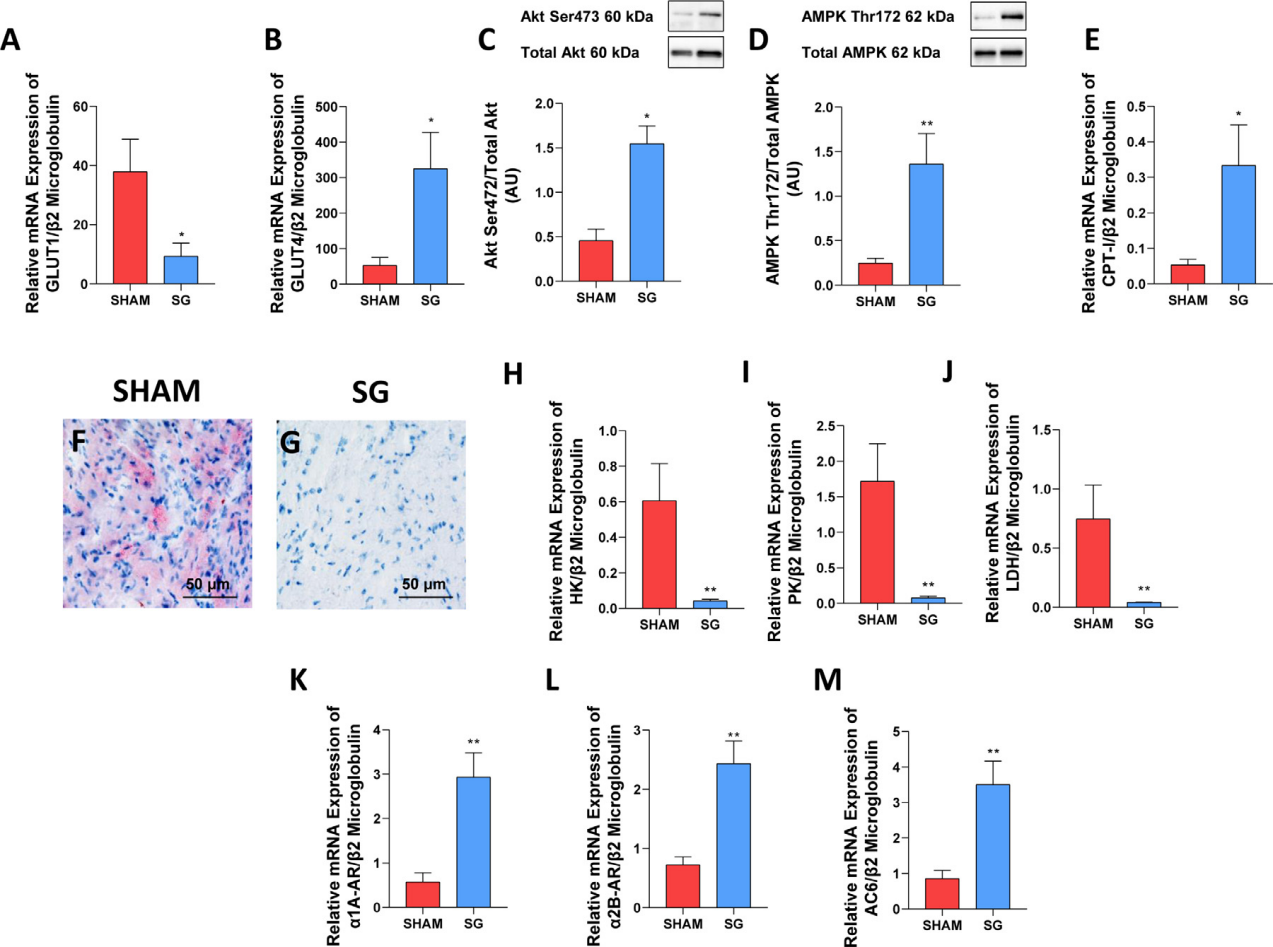
SG improves insulin signalling in rat myocardium. **Panel a,b:** GLUT1 (a) and GLUT4 (b) mRNA expression. SG was associated with 75% decrease and 513% increase in myocardial levels of total GLUT1 and GLUT4, respectively. **Panel c,d:** Akt phosphorylation on Ser473 (c) and AMPK phosphorylation on Thr172 (d) increased in the heart of SG rats when compared to sham-operated animals. **Panel e:** CPT-1 mRNA expression was significantly increased in the heart of SG rats when compared to sham-operated animals. **Panels f,g:** Oil red O staining of heart sections. SG rats (g) showed lower cardiac lipid accumulation compared to sham-operated animals (f). Magnification 20X. Scale bar: 50 μm. **Panels h–j:** mRNA expression of HK (h), PK (i) and LDH (j) was significantly decreased in the heart of SG rats when compared to sham-operated animals. **Panels k–m:** mRNA expression of a1-ARs 1a (k), 2b (l) and AC6 (m) was significantly increased in the heart of rats operated of SG when compared to sham-operated animals. GLUT1/4, Glucose transporter 1/4; AMPK, AMP-activated protein kinase; CPTI, Carnitine palmitoyltransferase I; HK, Hexokinase; PK, Pyruvate kinase; LDH, Lactate dehydrogenase; α1A-AR, Alpha-1A Adrenergic Receptor; α2B-AR, Alpha-2B Adrenergic Receptor; AC6, Adenylate Cyclase 6. ^⁎⁎^*P* < 0.008; *P < 0.050 (Student's *t*-test). Data are mean ± SEM (*n* = 5 rat per group).

SG was associated with 75% decrease and 513% increase in myocardial levels of total GLUT1 and GLUT4, respectively, the major myocardial glucose transporters (Figure 5 Panels a and b; Table S3). Since GLUT4 is rapidly upregulated after birth, GLUT4 represents the primary glucose transporter in the rat adult heart. Akt phosphorylation on Ser473 increased in the heart of SG with respect to sham-operated rats (Figure 5 Panel c) indicating improved insulin signaling and increased GLUT4 transmigration on cell membranes. In fact, it is widely accepted that Akt phosphorylation induces glucose uptake via translocation of the glucose transporter GLUT4 to the plasma membrane.^30^ Insulin resistance impairs Akt phosphorylation and reduces glucose uptake. In our animal model of dietary-induced obesity and insulin resistance, SG increased whole-body insulin-mediated glucose disposal, as shown by the glucose-AUC to insulin-AUC ratio compared with sham-operated rats.

Thr172 phosphorylation of AMPK was higher in the hearts of rats undergoing SG as compared with sham-operated ones (Figure 5 Panel d).

Once activated, AMPK inhibits protein synthesis,^31^^,^^32^ and enhances catabolic pathways, including glucose and fatty acid oxidation, to restore energetic balance.^33^

Enhanced fatty acid transportation in mitochondria and oxidative mitochondrial efficiency (Figure 5 Panel e) reduced myocardial fat depots after SG. Indeed, when fatty acid oxidation is impaired, as it occurs in insulin resistance, cells protect themselves from lipotoxicity by sequestering NEFA under form of triacylglycerol within lipid droplets.^34^^,^^35^

Furthermore, we observed reduced fat accumulation around the heart (epicardial fat: 0.75 ± 0.03 vs. 0.40 ± 0.03 g; *P* = 0.008 Holm-Bonferroni method) and a reduced lipid deposition (Figure 5 Panels f and g) after SG as compared with sham-operated rats.

SG also decreases glycolysis rate as shown by reduced gene expression of anaerobic glycolysis key enzymes compared to the sham operated group (Table S3) (Figure 5 Panels h–j).

We observed an increased expression of a1-ARs 1a and 2b (0.57 ± 0.21 vs.2.93 ± 0.54 Relative expression to β2 Microglobulin, *P* = 0.008 Student's *t*-test; 0.72 ± 0.14 vs.2.43 ± 0.38 Relative expression to β2 Microglobulin, *P* = 0.008 Student's *t*-test) as well as a raise in the expression of AC6 (0.86 ± 0.23 vs.3.51 ± 0.66 Relative expression to β2 Microglobulin, *P* = 0.008 Student's *t*-test) in the heart of rats operated of SG accounting for the improved myocardial function (Figure 5 Panels k–m).

#### *In vitro* experiments

To test if the beneficial effects of SG on heart metabolic state were linked to the decrease of BCAAs, we performed *in vitro* stimulation of rat primary cardiomyocyte cultures in the presence or absence of insulin (100 nM) and BCAAs (2.15 mM).

**Figure 6:**
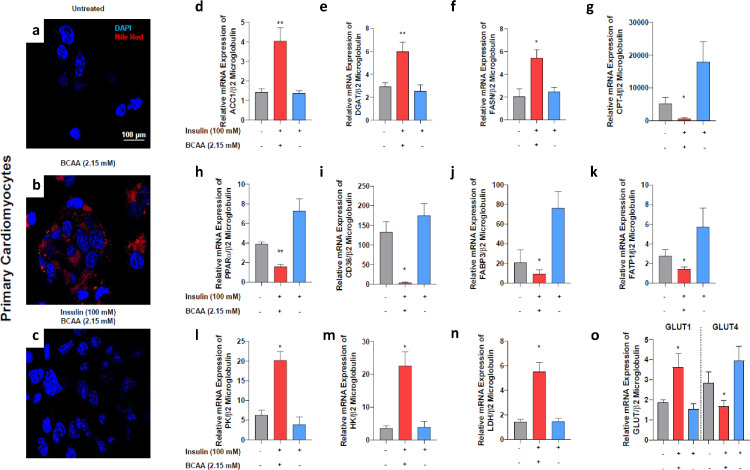
*In vitro* experiments. **Panels a,c:** Nile red staining of primary cardiomyocytes incubated with or without BCAA (2.15 mM) and in presence or absence of insulin (100 nM). Magnification 60X. Scale bar: 50 µm. **Panels d–h:** BCAA stimulation significantly decreased gene expression of PPARα (d) and Fatty acid transporters (e–h). **Panels i,j:** BCAAs stimulation increased gene expression of GLUT1 (i) and reduced that of GLUT4 (j). **Panels k–m:** BCAAs stimulation increased gene expression of glycolysis key enzymes HK (k), PK (l) and LDH (m). PPARα, Peroxisome proliferator-activated receptor α; GLUT1/4, Glucose transporter 1/4; CPTI, Carnitine palmitoyltransferase I; HK, Hexokinase; PK, Pyruvate kinase; LDH, Lactate dehydrogenase; ^⁎⁎^*P* = 0.002; *P < 0.050 (Student's *t*-test). Data in panels d–m are mean ± SEM of five independent experiments.

Stimulation of cardiomyocytes with insulin and BCAAs increased lipid droplet accumulation (Figure 6, Panels a–c), by increasing transcription factors involved in *de novo* lipogenesis and by decreasing those involved in fatty acid oxidation (Figure 6, Panels d–g, Table S4).

Moreover, insulin and BCAAs stimulation reduced fatty acid uptake and altered gene expression of glucose transporter, increasing GLUT1 and reducing GLUT4 expression, and raised glycolysis rate increasing gene expression of glycolysis key enzymes (Figure 6, Panels h–o, Table S4).

## Discussion

The main finding of our study was that at 1 year after SG there was a dramatic reduction of the baseline LVM. In fact, we found a 28% reduction of the LVM at 12 months following surgery that reached 43% at 48-month follow-up. SG in rats under a HFD induced a 35% reduction of the LVM and a significant improvement of the ejection fraction as compared with sham-operated ones. Although the effect on LVM reduction was significant after 6 months from SG, the patients had a rapid modification of the pattern of circulating metabolites that was appreciable already at 1 month following surgery. This suggest that SG plays a metabolic effect that goes beyond mere weight loss.

Then we sought to clarify the mechanism of action of SG on the reduction of LVM and, thus, we compared the effects of SG with those of a sham operation on BCAA, glucose and fatty acid metabolism as well as on insulin signalling in rats with diet-induced obesity.

We^36^ and other authors^37^^,^^38^ previously investigated the changes of LVM after bariatric surgery and found cardiac remodelling following surgery. A meta-analysis showed postsurgical reduction of LVM ranging between −16 g and −63 g, with a significant standardized mean difference of −0.46 (95% confidence interval (CI) = 0.36–0.56; *P* < 0.001). In the present study, we found an even more prominent effect of SG with a reduction of LVM of 30 g on average at 6 months, of 71 g at 12 months and 108 g at 48 months following SG. This difference can arise from the different surgical operations or technique performed in the various studies analysed in the meta-analysis.

Previous studies with low-calorie diets demonstrated a small reduction of LVM. A 6-month hypocaloric diet, poor in carbohydrates or in fat, induced a decrease in body weight of 6,7 kg and of about 5 g in the LVM (*P* < 0.001) in each group compared with baseline.^7^

The EMPA-HEART CardioLink-6 trial showed that inhibiting SGLT2 with empagliflozin was accompanied with a significant reduction in LVM in patients with type 2 diabetes mellitus and coronary artery disease.^39^

The STAMPEDE (Surgical Treatment and Medications Potentially Eradicate Diabetes Efficiently) trial showed in the long run positive changes of cardiometabolic biomarkers related to lipids, inflammation, thrombosis, and obesity.^40^

In our study, the major determinants of LVM were weight, epicardial fat, metabolomic BCAAs and systolic blood pressure.

Epicardial fat thickness was reduced by 24% at 12 months and halved at 48 months following surgery, becoming similar to the values observed in healthy lean subjects.^41^

Metabolomic profile in humans closely resembled that observed in rodents with reduction of BCAAs, ketone bodies and lactate after bariatric surgery. Notably, in humans BCAAs levels fell down already at 1 month after SG.

We studied the protein expression of the two major enzymes that catabolize BCAA in the heart and found a significantly higher expression of both BCAT2 and BCKDH in rats undergone SG versus sham operation. BCAT2 catalyses the transamination of BCAAs to their respective alpha-keto acids (KA) while the BCKDH enzyme complex coverts BCKAs into branched-chains acyl coenzyme A that are oxidized in the mitochondria. Our data suggest that SG reduces BCAA overload and improves cardiac BCAA utilization.

In obesity, the suppression of BCAA and BCKA catabolism in the adipose tissue and in the liver causes a chronic increase of their circulating levels.^42^ Elevated levels of cardiac BCKA promote protein synthesis and cardiac hypertrophy associated with impaired cardiac contractility.^43^ Furthermore, compensatory ventricular hypertrophy in early stages of heart failure associates with increased plasma levels of both BCAA and BCKA.^44^

Compared with sham-operated rodents, we observed a net increment of myocardial expression of enzymes involved in fatty acid and glucose metabolism as well as key enzymes of the insulin-signalling pathway, after SG. Consequently, a reduced myocardial lipid accumulation was observed, with reduced lipotoxicity that, together with high levels of phosphorylated AMPK, contribute to the reversal of cardiac hypertrophy.

Increased expression of alpha1 adrenergic receptors (a1-ARs) 1a and 2b as well as a raise in the expression of adenylyl cyclase type 6 (AC6) in the heart of rats operated of SG account for the improved myocardial function. In fact, long-term activation of cardiac α1-ARs counteracts the negative effects of overstimulation of β1-ARs in heart failure,^45^ while AC6 is necessary for calcium handling and myocardium contractility.^46^

We demonstrated a positive effect of BCAAs on lipid accumulation in primary cultures of rat cardiomyocytes. Expression of fatty acid translocase (FAT/CD36), fatty acid transport proteins (FATP1-6) and plasma membrane fatty acid binding protein (FABPpm), identified as putative fatty acid transporters, decreased in the presence of BCAAs. However, lipids accumulation in cardiomyocytes resulted from the combination of an increase in *de novo* lipogenesis and a reduction of fatty acid oxidation, as shown by the reduced expression of carnitine-palmitoyltransferase I (CPT-I).

Moreover, BCAAs increased the expression of GLUT1 and reduced that of GLUT4.

Increased expression of GLUT1 and reduced expression of GLUT4, which is the predominant isoform of glucose transporter present in the normal myocardium, has been observed in post-ischemic reperfusion,^47^ post-infarction heart failure^48^ and myocardial hypertrophy.^49^ However, the increased expression of GLUT1 in hypertrophied hearts did not result in significant changes in expression of glycolytic enzymes. A major modification of the glucose metabolism during cardiac hypertrophy is, in fact, enhanced glycolysis likely vicariting an impaired fatty acid oxidation through the Randle cycle.^50^

Therefore, increased levels of circulating BCAAs seem to cause cardiac metabolic alterations with compensatory myocardial hypertrophy. SG counteracts obesity-linked cardiac injury by reducing BCAAs plasma levels and by inducing weight loss with reduced cardiac overload.

Limitations of our study include the relatively small number of participants, although the study was sufficiently powered, and its single centre nature. Although the sample size was limited, the statistical analysis confirmed it was sufficiently powered. Demographic data showed that patients had characteristics, which are generalizable with respect to that of the standard population undergoing bariatric/metabolic surgery.

Strengths of our investigation are the long and frequent follow-up with 100% retention of the participants and the use of a rodent model of sleeve gastrectomy in diet-induced obesity and *in vitro* mechanistic studies. The translational approach allowed to comprehensively demonstrate the improvement of cardiac metabolism, reducing potential bias.

In conclusion, after SG a sustained and long-lasting weight loss with a remarkable and prompt decrease of the left ventricular mass, epicardial fat and insulin resistance. Animal and *in vitro* studies suggest a net improvement of cardiac BCAA metabolism together with the amelioration of fat oxidation and insulin signaling and help understanding how SG reduces intra-myocytic fat accumulation and lipotoxicity. However, further detailed mechanistic studies are warranted.

## Contributors

LCG, GC, GM and NB designed the study. EC and GM did echocardiograms blinded analysis. LCG, NB and GA performed the animal studies. LT, CLi and CLu performed metabolomics both in human and in animal samples. GA did molecular biology assays. ALT measured gene expression. LCG, GC, GM, NB, GA and SRB wrote the manuscript. DLB substantially contributed to the interpretation and discussion of the results. LCG, GA, GC, GM verified the underlying data All authors provided critical feedback and helped shape the research, analysis and manuscript. The authors declare that the data supporting the findings of this study are available within the paper. GC is the guarantor of this work and, as such, had full access to all the data in the study and takes responsibility for the integrity of the data and the accuracy of the data analysis. All authors read and approved the final version of the manuscript.

### Data sharing statement

Metabolomics raw data is deposited on the online repository Figshare with the following digital object identifier: 10.6084/m9.figshare.18550760. The full uncropped blots are provided as “Supplementary Full Blots” in the manuscript. All the other data and materials supporting the results or analyses presented in this paper available upon reasonable request.

## Declaration of interests

All authors report no conflict of interest.

## Appendix Supplementary materials

Image, application 1

Image, application 2

Image, application 3
