# Supplementary figures and images for: The early reduction of left ventricular mass after sleeve gastrectomy depends on the fall of branched-chain amino acid circulating levels
Source: eBioMedicine. 2022 Feb 4;76:103864. doi: 10.1016/j.ebiom.2022.103864 (PMC8829082; doi:10.1016/j.ebiom.2022.103864)

**Figure 3 Panel C**

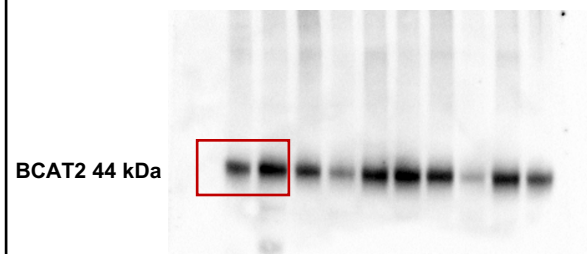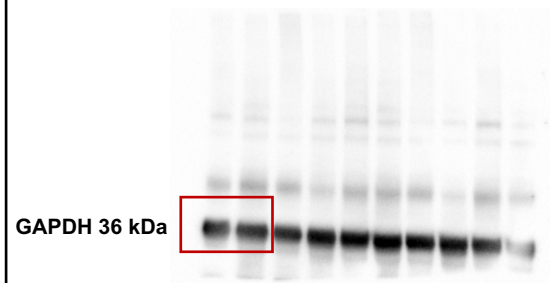

**Figure 3 Panel D**

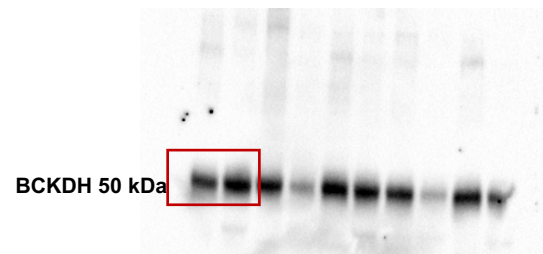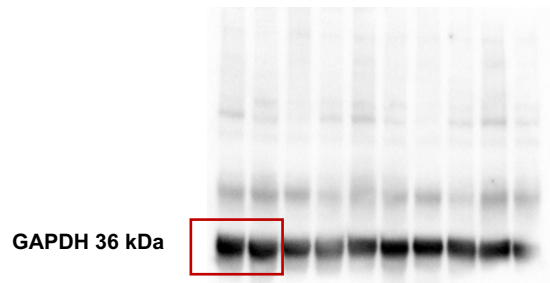

**Figure 4 Panel E**

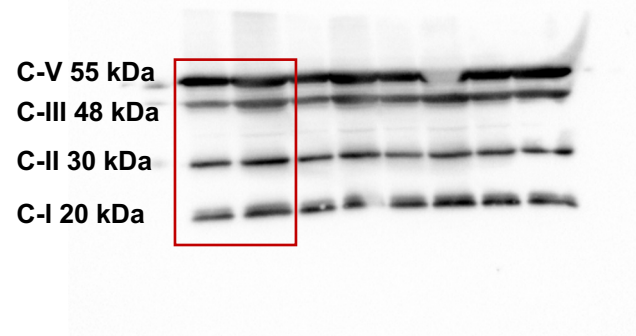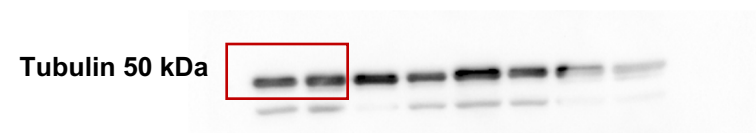

**Figure 5 Panel C**

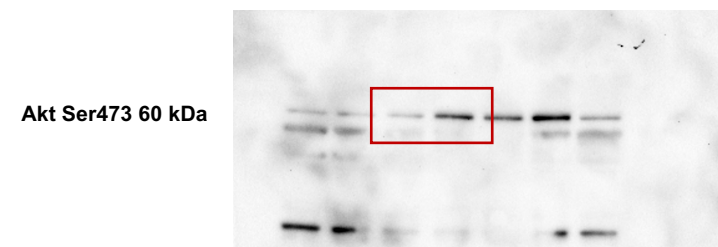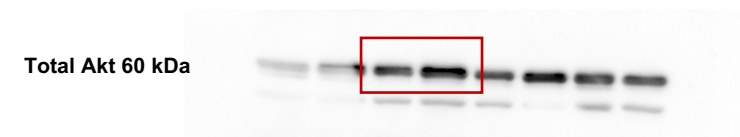

**Figure 5 Panel D**

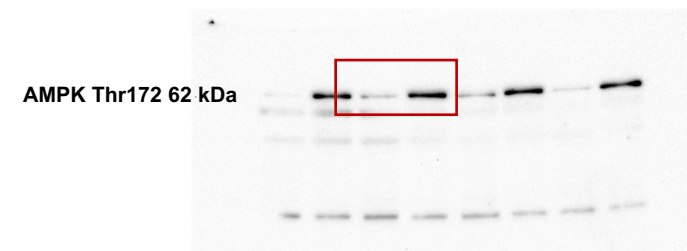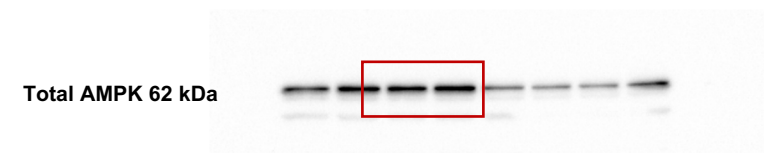

Supplement: Supplementary file 1 [file mmc1.pdf]
