# Supplementary material for: The early reduction of left ventricular mass after sleeve gastrectomy depends on the fall of branched-chain amino acid circulating levels
Source: eBioMedicine. 2022 Feb 4;76:103864. doi: 10.1016/j.ebiom.2022.103864 (PMC8829082; doi:10.1016/j.ebiom.2022.103864)

**SUPPLEMENTARY FIGURE LEGENDS**

**Supplementary Figure 1. Sleeve Gastrectomy (SG) is associated with a net reduction of LVM and epicardial fat thickness.**

**Panel a**: Box plots of left ventricular mass changes. The left ventricular mass decreased consistently until 48 months following SG.

**Panel b:** Box plots of epicardial fat thickness changes. Epicardial fat thickness constantly decreased over time and halved at 48 months after SG.

**
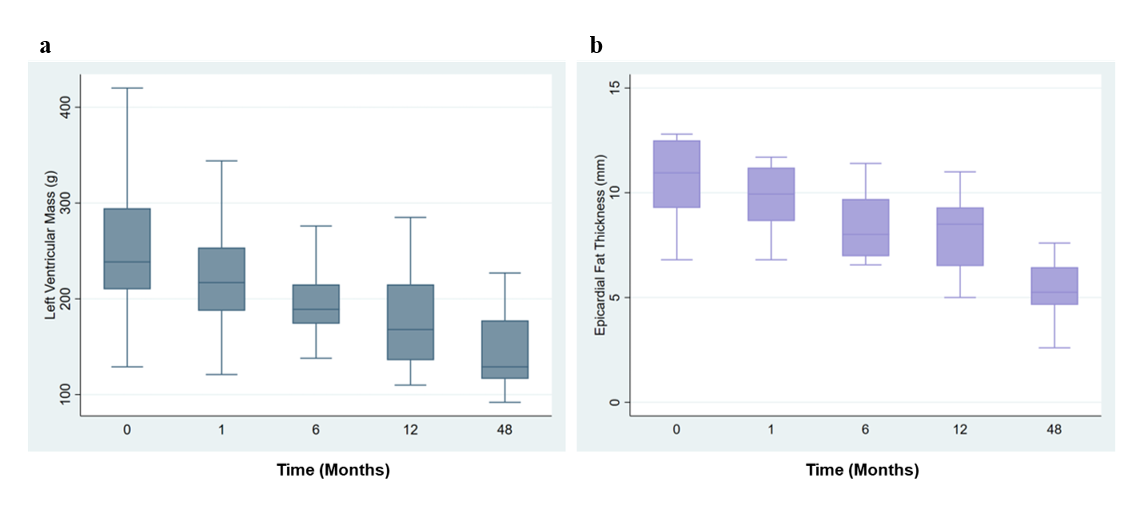
**

**Supplementary Figure 2. VIP and metabolites that correlate with lactate.**

**Panel a**: Important human metabolites selected based on VIP score. After Sleeve Gastrectomy, 43% of the metabolites changed significantly, but only four of them had a VIP score >1.

**Panel b**: Metabolites correlation with lactate. Pyruvate, alanine and 3-OH-butyrate displayed the highest correlations.

VIP, Variable importance in the projection.


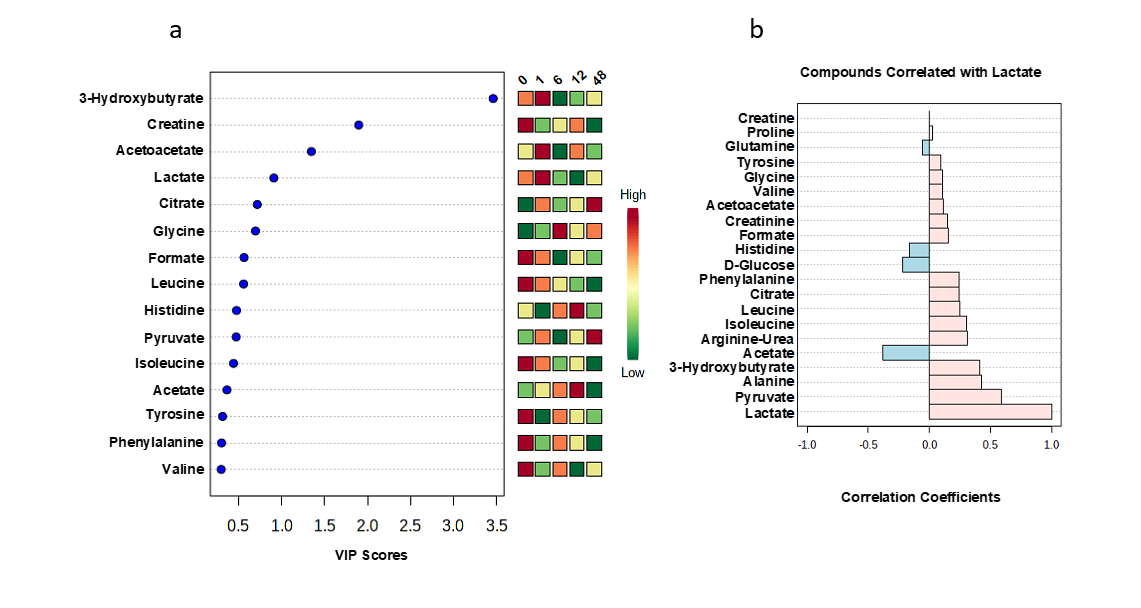


**Supplementary Figure 3. Important rodent metabolites selected based on VIP score.**

After SG, 25% of the metabolites changed significantly and nine had a VIP score >1.

VIP, Variable importance in the projection.


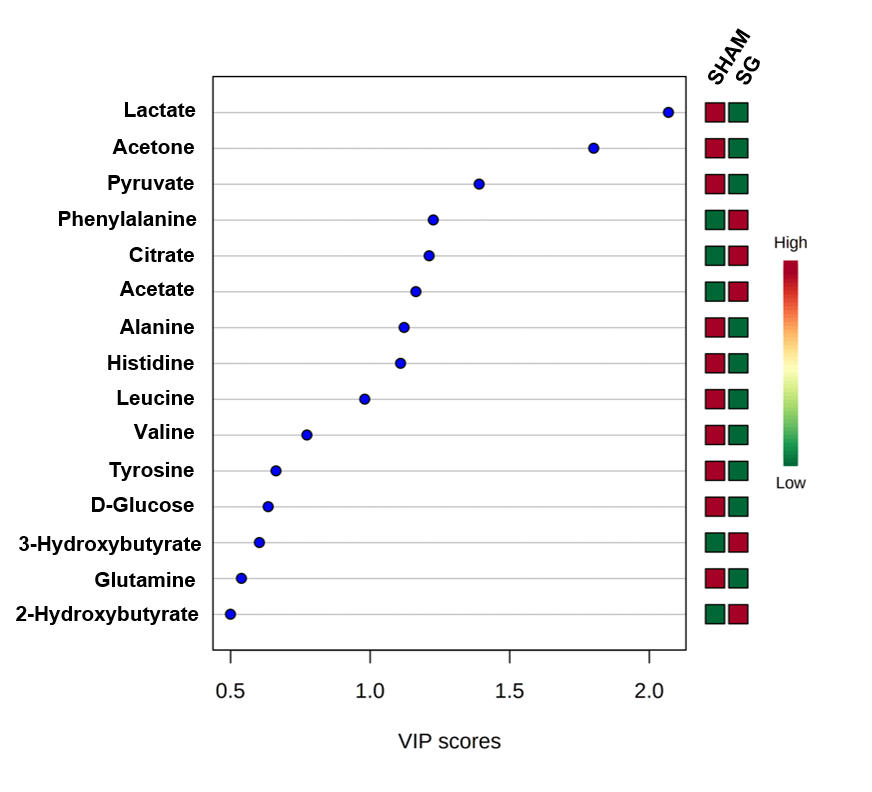

Supplement: Supplementary file 2 [file mmc2.docx]
