# Supplementary material for: The early reduction of left ventricular mass after sleeve gastrectomy depends on the fall of branched-chain amino acid circulating levels
Source: eBioMedicine. 2022 Feb 4;76:103864. doi: 10.1016/j.ebiom.2022.103864 (PMC8829082; doi:10.1016/j.ebiom.2022.103864)
Supplement: Supplementary file 3 [file mmc3.docx]

| Gene | Primer sequence (5′to 3′) |
| --- | --- |
| Peroxisome proliferator-activated receptor alpha (PPARα) | TGGCGTTCGCAGCTGTTTTG |
|  | CCACAGAGCACCAATCTGTGATG |
| Carnitine palmitoyltransferase 1 (CPT-1) | TTCAGGAGCTGGATGGCTTG |
|  | CCCTTGGGGTCATTTGGTGA |
| Fatty-acid-binding protein 1 (FABP1) | GCAAGTACCAAGTGCAGAGC |
|  | TGCACGATTTCTGACACCCC |
| Hexokinase-1 (HK1) | CTCCGAACCACTCAAGACCC |
|  | ACATCTGCAGTAGACGCACC |
| Pyruvate kinase (PK) | CTTCCCCTTGCTCTACCGTG |
|  | ACCACGGAGCTTTCCACTTTC |
| Glucose transporter 1 (GLUT1) | CGGGCATCAATGCTGTGTTC |
|  | GGCACTAGGCCAATCAAGGT |
| Glucose transporter 4 (GLUT4) | TCCAGTATGTTGCGGATGCT3 |
|  | CCTCCCCGAAAAGTCAGTCC |
| Lactate dehydrogenase (LDH) | TTGTCTGGACAAGATGGCAAC |
|  | TGCCGTACATTCCCTTCACC |
| Alpha-1A adrenergic receptor (α1A-AR) | TCATGGGCCTCTGCATCATC |
|  | AATCCGGGAAGAAAGACCCAAT |
| Alpha-2B adrenergic receptor (α2B-AR) | AGTCAGTTCTGTGCGTCCTG |
|  | GTAGCCACTAGGATGTCGGC |
| Acetyl-CoA Carboxylase (Acc1) | CACCAAGAATGCCCCTGCC |
|  | CGTTAGCACCGAGCTGCTTC |
| Fatty acid synthase (Fasn) | GAATCCGCACAGGCTACCAA |
|  | CTGGGCTTCACCATCACCAT |
| Diglyceride acyltransferase (Dgat) | AGCAGGAGTAGGCCCCATAG |
|  | ATTGGGGCTTACCTTGTCCG |
| Diglyceride acyltransferase (Dgat) | AGCAGGAGTAGGCCCCATAG |
|  | ATTGGGGCTTACCTTGTCCG |
| Adenylyl cyclase type 6 (AC6) | CACCAAGAATGCCCCTGCC |
|  | CGTTAGCACCGAGCTGCTTC |
| β2-microglobulin | GACCGTGATCTTTCTGGTGCT |
|  | ACACTTGAATTTGGGGAGTTTTCTG |

**Table S1.** Primer sequences used in qRT-PCR

mRNA expression levels were normalized to Beta2 microglobulin and quantification of relative gene expression was calculated using the 2-∆CT (comparative threshold) method.

| Variables | Baseline | Months After Sleeve Gastrectomy | | | |
| --- | --- | --- | --- | --- | --- |
|  |  | **1 month** | **6 months** | **12 months** | **48 months** |
| Beta-blocker | 10 (50%) | 10 (50%) | 10 (50%) | 10 (50%) | 10 (50%) |
| Calcium channel blocker | 20 (100%) | 6 (30%) | 6 (30%) | 4 (20%) | 4 (20%) |
| ACE-inhibitor or ARB | 10 (50%) | 9 (45%) | 9 (45%) | 9 (45%) | 9 (45%) |
| Diuretics | 13 (65%) | 6 (30%) | 0 (0%) | 0 (0%) | 0 (0%) |
| Lipid lowering agents | 14 (70%) | 7 (35%) | 5 (25%) | 5 (25%) | 2 (10%) |
| Biguanides | 10 (50%) | 10 (50%) | 10 (50%) | 8 (40%) | 7 (35%) |
| TZD | 1 (5%) | 0 (0%) | 0 (0%) | 0 (0%) | 0 (0%) |
| GLP1 agonists | 8 (40%) | 3 (15%) | 3 (15%) | 1 (5%) | 0 (0%) |
| SGLT2 inhibitors | 5 (25%) | 2 (10%) | 2 (10%) | 0 (0%) | 0 (0%) |
| insulin | 4 (20%) | 0 (0%) | 0 (0%) | 0 (0%) | 0 (0%) |
| Average number of antihypertensive drugs per day | 3.25±1.02 | 1.55±0.76* | 1.25±0.55* | 1.15±0.49^#^ | 1.15±0.49^#^ |
| Average number of antihyperglycemic drugs per day | 1.95±2.01 | 1.30±1.38* | 1.30±1.38* | 0.80±1.06^#^ | 0.60±0.88^#^ |

**Table S2**. Medication data at baseline and at 1, 6, 12 and 48 months after Sleeve Gastrectomy.

Categorical variables are summarized with the use of frequencies. Significance were calculated by repeated measure ANOVA. ^*^ P<0.01 ^#^ P<0.0001.

ACE, Angiotensin-converting enzyme; ARB, Angiotensin II receptor blockers; TZD, Thiazolidinediones; GLP1, Glucagon-like peptide 1; SGLT2, Sodium–glucose cotransporter 2.

|  | Sham-operation | Sleeve gastrectomy |
| --- | --- | --- |
| Quantitative Real Time PCR | | |
| PPARα (Relative Expression) | 0.44±0.10 | 1.98±0.35 |
| P |  | 0.003 |
| CPTI (Relative Expression) | 0.05±0.01 | 0.33±0.11 |
| P |  | 0.041 |
| HK (Relative Expression) | 0.61±0.21 | 0.04±0.001 |
| P |  | 0.008 |
| PK (Relative Expression) | 1.72±0.08 | 0.08±0.02 |
| P |  | 0.008 |
| CD36 (Relative Expression) | 2.22±0.57 | 9.09±1.48 |
| P |  | 0.008 |
| FATP1(Relative Expression) | 0.91±0.03 | 1.31±0.05 |
| P |  | 0.008 |
| FABP (Relative Expression) | 1.37±0.34 | 6.37±1.35 |
| P |  | 0.016 |
| GLUT1 (Relative Expression) | 37.95±10.93 | 9.39±4.46 |
| P |  | 0.032 |
| GLUT4 (Relative Expression) | 53.07±22.26 | 325.30±101.9 |
| P |  | 0.016 |
| LDH (Relative Expression) | 0.75±0.29 | 0.04±0.001 |
| P |  | 0.008 |
| α1A-AR (Relative Expression) | 0.57±0.21 | 2.93±0.54 |
| P |  | 0.008 |
| α2B-AR (Relative Expression) | 0.72±0.14 | 2.43±0.38 |
| P |  | 0.008 |
| AC6 | 0.86±0.23 | 3.51±0.66 |
| P |  | 0.008 |
| Western Blot Analysis | | |
| C-I (AU) | 1.56±0.08 | 3.70±0.57 |
| P |  | 0.008 |
| C-II (AU) | 1.74±0.05 | 4.00±0.05 |
| P |  | 0.008 |
| C-III (AU) | 2.39±0.35 | 5.90±0.33 |
| P |  | 0.008 |
| C-V (AU) | 3.04±0.27 | 5.19±0.70 |
| P |  | 0.032 |
| pAkt Ser472 (AU) | 0.46±0.13 | 1.55±0.20 |
| P |  | 0.016 |
| pAMPK Thr172 (AU) | 0.25±0.05 | 1.36±0.34 |
| P |  | 0.008 |

**Table S3.** Real Time and Western blot analyses

**Upper section**: mRNA expression key enzymes of fatty acid oxidation, glycolysis and glucose transporters.

PPARα, Peroxisome proliferator-activated receptor α; CPTI, Carnitine palmitoyltransferase I; HK, Hexokinase; PK, Pyruvate kinase; CD36, Cluster of differentiation 36; FATP1, fatty acid transport protein 1; FABP, Fatty Acid-Binding Protein; GLUT1/4, Glucose transporter 1/4; LDH, Lactate dehydrogenase; α1A-AR, Alpha-1A Adrenergic Receptor; α2B-AR, Alpha-2B Adrenergic Receptor; AC6, Adenylate Cyclase 6.

**Lower section**: protein expression of oxidative phosphorylation system and AMPK phosphorylation.

C-I/II/III/V, Complex I/II/III/V; AMPK, AMP-activated protein kinase.

|  | Untreated | BCAA+Insulin | Insulin |
| --- | --- | --- | --- |
| Quantitative Real Time PCR | | | |
| PPARα (Relative Expression) | 3.88±0.23 | 1.60±0.23 | 7.28±1.24 |
| P |  | 0.002 |  |
| CPTI (Relative Expression) | 5277±1914 | 654.0±352.1 | 17942±6187 |
| P |  | 0.004 |  |
| HK (Relative Expression) | 3.53±0.85 | 22.54±4.29 | 3.91±1.73 |
| P |  | 0.014 |  |
| PK (Relative Expression) | 6.26±1.27 | 20.20±2.22 | 3.89±1.94 |
| P |  | 0.006 |  |
| CD36 (Relative Expression) | 132.7±26.86 | 4.01±1.84 | 174.40±30.93 |
| P |  | 0.024 |  |
| FATP1(Relative Expression) | 2.76±0.65 | 1.42±0.24 | 5.73±1.93 |
| P |  | 0.037 |  |
| FABP (Relative Expression) | 20.85±12.99 | 9.24±4.02 | 76.24±16.88 |
| P |  | 0.043 |  |
| GLUT1 (Relative Expression) | 1.86±1.35 | 3.63±0.67 | 1.54±0.27 |
| P |  | 0.008 |  |
| GLUT4 (Relative Expression) | 2.84±0.55 | 1.67±0.29 | 3.94±0.73 |
| P |  | 0.008 |  |
| LDH (Relative Expression) | 1.43±0.21 | 5.50±0.78 | 1.45±0.29 |
| P |  | 0.043 |  |
| ACC1 (Relative Expression) | 1.43±0.18 | 4.04±0.69 | 1.37±0.13 |
| P |  | 0.008 |  |
| FASN (Relative Expression) | 2.04±0.69 | 5.44±0.73 | 2.47±0.40 |
| P |  | 0.045 |  |
| DGAT (Relative Expression) | 2.91±0.33 | 5.96±0.84 | 2.53±0.55 |
| P |  | 0.008 |  |

**Table S4.** In vitro Experiments

mRNA expression key enzymes of fatty acid transport and oxidation, glycolysis, glucose transporters and de novo lipogenesis.

PPARα, Peroxisome proliferator-activated receptor α; CPTI, Carnitine palmitoyltransferase I; HK, Hexokinase; PK, Pyruvate kinase; CD36, Cluster of differentiation 36; FATP1, fatty acid transport protein 1; FABP, Fatty Acid-Binding Protein; GLUT1/4, Glucose transporter 1/4; LDH, Lactate dehydrogenase; ACC1, Acetyl-CoA carboxylase; FASN, Fatty acid synthase; DGAT, Diglyceride acyltransferase.
